# High-throughput phenotyping of wheat ear surface area and ear density in the field

**DOI:** 10.1016/j.plaphe.2026.100199

**Published:** 2026-03-09

**Authors:** Marie-Pia D'Argaignon, Raul Lopez-Lozano, Sylvain Jay, Aurélien Ausset, Bruno Berthon, Philippe Burger, Romain Chapuis, Benoît de Solan, Antonin Grau, Florian Larue, Romane Le-Roy, Rémy Marandel, Vincent Mercier, Mathieu Roy, Gilles Tison, Frédéric Venault, Pierre Martre

**Affiliations:** a EMMAH, INRAE, Avignon Université, Avignon, France; b LEPSE, Univ. Montpellier, INRAE, Institute Agro Montpellier, Montpellier, France; c Diascope, Univ Montpellier, INRAE, Mauguio, France; d Arvalis Institut du Végétal, Avignon, France; e APC, INRAE, Auzeville, France

**Keywords:** RGB images, Deep learning, Ear area index, Yield components, Phenomobile

## Abstract

Ear density ($D_{e}$) and ear surface area $\left(S_{e}\right)$ in cereals are important traits for adaptation to low inputs and climate change. Here we propose a high-throughput field phenotyping method to estimate these traits using nadir and 45° RGB images acquired by the Phenomobile ground robot. First, the YOLOv5 ear detection algorithm is applied to nadir RGB images to estimate $D_{e}$. Second, an ear segmentation algorithm is applied to nadir and 45° RGB images to compute the ear gap fraction at different viewing angles. The Beer-Lambert law is then inverted to compute the ear area index (EAI) from the observed ear gap fraction. $S_{e}$ is finally derived as the ratio between EAI and $D_{e}$.

We applied the methodology to a panel of 10 commercial bread wheat varieties to analyse how both traits vary across 12 environments. The relative error obtained for awnless varieties is 12% (56 ears m^−2^) for $D_{e}$ and 18% (1.3 cm^2^) for $S_{e}$. For awned varieties, ground-truth observations of $S_{e}$ were shown to be biased due to an overestimation of awns contribution, leading to an error of 41% (3.6 cm^2^). $S_{e}$ was strongly correlated with grain dry mass per ear at harvest (*r*^*2*^ = 0.80 across genotypes and environments, *r*^*2*^ per genotype ranged between 0.80 and 0.95) and EAI was strongly correlated with grain yield (*r*^*2*^ = 0.83). These results indicate that both EAI and $S_{e}$ can be interesting non-destructive proxies for yield and grain dry mass per ear.

## 1 Introduction

Wheat is one of the three most important staple crops, with maize and rice [1] and its consumption will continue to increase in line with the population growth. In the same time, natural conditions are changing, and the natural resources are diminishing [2]. In many regions of the world the grain yield has not progressed in the last two decades [3,4]. In this context, breeding for new, improved varieties adapted to future climate conditions and to low input systems is an important pillar to satisfy the increasing global food demand.

The development in the last decade of high-throughput field phenotyping platforms and instruments has opened a new era in plant phenomics [5]. Recent works have developed algorithms to achieve reliable estimations at high throughput of different wheat morphological traits that are relevant to characterize the functioning of wheat genotypes such as plant height [6], fractional vegetation cover [7], or the plant density at emergence [8,9].

The implementation of deep learning models in plant phenotyping opened the possibility to achieve estimation of new traits related with the detection or counting of individual plant organs from RGB images [10].Ears counting has been one of the tasks that has concentrated more attention [[11], [12], [13]] since ear density ($D_{e}$, ears/m^2^), the number of ears by square meter, constitutes an essential yield component, highly relevant for breeding, but time-consuming to manually estimate in the field. The international Global Wheat Head Detection (GWHD) challenge launched in 2020 permitted the phenotyping community to improve the reliability and generalization of convolutional neural networks (CNN), a category of deep learning models for ear counting and $D_{e}$ estimation [14,15].

Besides $D_{e}$, ear morphological traits including its length and compactness are also heritable traits [16] that are linked to wheat yield potential [17]. However, the use of deep learning techniques like CNN to estimate such kind of traits have received less attention. Some studies have shown promising results to count the number of spikelets per ear on RGB images taken in the laboratory [18,19] but not yet in actual field conditions where ear overlapping and the effect of the ears background in the RGB images can represent a challenge for such algorithms. Recent studies have begun to evaluate the use of three-dimensional reconstruction techniques from two-dimensional RGB images, such as neural radiance fields (NeRFs) or Gaussian splatting, to model canopy structure [20]. In particular, Gaussian splatting shows potential for measuring wheat ear length, width and volume [21] from 3D point clouds generated using multi-view RGB images. However its accuracy can be reduced in complex canopy structures, for example in high ear density conditions where instance segmentation of 3D point clouds may present some inaccuracies to identify correctly the ears and determine their dimensions. Although very promising for organ-level reconstruction in field conditions, the use of these techniques for ear area or volume estimation still needs to be fully analysed and evaluated.

The aim of the present study was to estimate $D_{e}$ and the average ear surface area ($S_{e}$), defined as the average vertical cross-sectional area (in cm^2^) of an ear, across wheat microplots for high-throughput phenotyping applications. To do so, we propose a methodology that combines CNN applied to RGB images with the Beer-Lambert law (BL). The BL relates light interception by a turbid medium with the size or area of its scattering elements, and is commonly used in optics to estimate leaf area index (LAI) of plant canopies from light transmission measured with an optical device. [22]. The proposed methodology is applied in two different steps. First, a CNN for ear detection is applied to estimate $D_{e}$. Second, ears gap fraction, calculated from masks generated by a CNN for ear segmentation, is then used to solve the BL equation and to estimate the canopy ear area index (EAI) that, divided by $D_{e}$, allows us to derive $S_{e}$. The methodology proposed is evaluated in a multi-environment wheat trial. This paper addresses the following research questions: (i) what is the accuracy and heritability of the proposed methodology to estimate both $D_{e}$ and the $S_{e}$ in wheat microplots? (ii) are these methods able to describe the variability of both traits linked to genotype by environment interactions (GxE)? (iii) can the estimates of the $S_{e}$ serve as a proxy for yield components, such as the number of grains or the grain dry mass per ear?

## 2 Materials and methods

### 2.1 Study sites

**Fig. 1:**
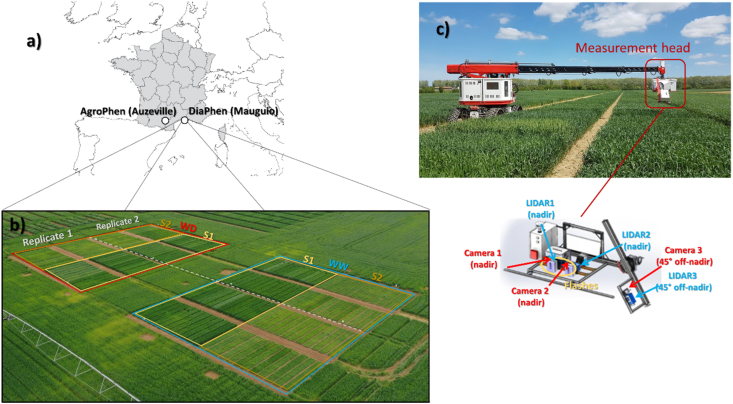
a) Map showing the location of the AgroPhen and DiaPhen field phenotyping platforms where field trials were conducted in this study. b) Aerial picture of the phenotyping trial at the DiaPhen platform in 2023 (Treatments: WD, rainfed; WW, irrigated; S1, autumn sowing; S2, winter sowing). c) Picture of the Phenomobile V2 with schema of the measurement head with the position of the three-LiDAR and three-RGB camera system and the flashes.

Field trials were conducted in DiaPhen field phenotyping platform in Mauguio, France (43°36ʹ46’’N, 03°58ʹ40’’E) during the 2021-2022 and 2022-2023 growing seasons (thereafter DiaPhen 2022 and DiaPhen 2023, respectively) and in AgroPhen field phenotyping platform platform in Auzeville, France (43°31′45’’N, 1°30′12’’E) during the 2022-2023 growing (thereafter AgroPhen 2023; Fig. 1).

All trials used a two-factor split-plot design with 80 microplots (Fig. 1b) and two replicates. The microplots were 10 m x 8 rows with a row spacing of 0.17 m at DiaPhen, and 10 m x 10 rows with a row spacing of 0.16 m at AgroPhen. Ten French bread wheat (*Triticum aestivum* L.) varieties (Supplementary Table S1), exhibiting variability in architectural traits (leaf stature, plant height, tillering capacity), and precocity, where grown in all trials. Five of these varieties are awned (Table S1). Two management factors were considered in each site: the sowing date and water availability at the DiaPhen platform; and the sowing date and sowing density at the AgroPhen platform. Soil water potential was monitored in the DiaPhen site using Watermark sensors (Irrometer Inc, US). In the well-watered treatments (WW) irrigation was applied regularly to keep soil water potential higher than −0.1 MPa. In the water deficit treatments (WD) irrigation was only applied to prevent severe water stress. Crop management practices including weed, disease and pest control, and nitrogen, potassium, phosphate and sulphur fertilizers were applied to prevent nutrients, weeds, diseases and pests from limiting crop growth and grain yield. N fertilizer was applied as ammonium nitrate in two (both years at DiaPhen and at AgroPhen for the sowing treatment S1) or three (at AgroPhen for the sowing treatment S2) split applications each growing season (Table 1).

**Table 1:** Sowing date, fertilizer rate, total irrigation, and cumulative precipitation, cumulative solar radiation, and average temperature from sowing to harvest.

Trial	Sowing date treatment^a^	Water supply or sowing density treatment^b^	Sowing date	Sowing density (seeds m^−2^)	N fertilizer rate (kg N ha^−1^)	Total irrigation (mm)	Cumulative precipitation (mm)	Cumulative solar radiaton (MJ m^−2^)	Average temeprature (°C)
DiaPhen 2022	S1	WW	Nov. 19, 2021	350	120	146	179	3308	13.2
		WD	Nov. 19, 2021	350	120	47	179	3308	13.2
	S2	WW	Jan. 14, 2022	350	120	162	113	3160	15.2
		WD	Jan. 14, 2022	350	120	45	113	3160	15.2
DiaPhen 2023	S1	WW	Nov. 7, 2022	350	113	159	313	3362	13.3
		WD	Nov. 7, 2022	350	113	29	313	3362	13.3
	S2	WW	Jan. 16, 2023	350	113	157	159	3116	14.6
		WD	Jan. 16, 2023	350	113	32	159	3116	14.6
AgroPhen 2023	S1	D1	Nov. 08, 2022	200	194	0	531	3028	12.5
		D2	Nov. 08, 2022	400	194	0	531	3028	12.5
	S2	D1	Dec. 7, 2022	200	140	0	432	2977	13.3
		D2	Dec. 7, 2022	400	140	0	432	2977	13.3

a S1, autumn sowing; S2, Winter sowing.

b WW, irrigated; WD, rainfed; D1, low sowing density; D2, heigh sowing density.

### 2.2 Field measurements of ear density, ear surface area and yield

At ripeness maturity, a sampling area consisting of four rows over a distance of 1 m was delineated in the central part of each plot. Stems were cut at the ground level and all plant material in the sampling area was collected and placed in labelled paper bags. In the laboratory, the fresh mass of the plant samples was measured with a precision scale. Then, a subsample of 15% (DiaPhen 2022) or 30% (DiaPhen and AgroPhen 2023) of the whole sample fresh mass was randomly selected, representing around 110 shots for a 30% sub-sample. The number of ears in the 30% (15%) subsample was then counted ($N_{e,30}$) and their one-sided projected area ($S_{e,30}$, cm^2^) was measured using a LiCOR-3100 planimeter (LI-COR Inc., Lincoln, NE, USA). The projected area measured by the LiCOR-3100 corresponds to the vertical cross-section of ears. Ten fertile shoots per sample were also randomly selected to measure with a ruler the ear length.

All sampled plant material were dried in a forced-air oven for 48 h at 80 °C, and both the total dry mass of the whole sample ($B_{t}$, g) and the subsample dry mass ($B_{30}$, g) were measured. The ears of the subsample were then threshed, and the dry mass of the grains was measured after drying in a forced-air oven for 48 h at 80 °C ($W_{g,30}$, g). The number of grains in sub-sample ($G_{e,30}$) was counted using a CONTADOR seed counter (PFEUFFER GmbH, Kitzingen, Germany) and the thousand kernel weight (TKW, g) was determined as the ratio of $W_{g,30}$ to $G_{e,30}$.

The ear density ($D_{e}$, ears m^−2^) was retrieved from the number of ears in the subsamples adjusted by the fraction of the dry mass of the subsamples and then divided by the surface area of the sample:

(1) $$D_{e}=\frac{N_{e,30}*B_{t}/B_{30}}{4l*d_{r}}$$

where 4 is the number of rows sampled, l (=1m) is the sampling length and $d_{r}$ (m) is the row spacing.

The average ear surface area $(S_{e}$, cm^2^), expressed as the one-sided projected area of the vertical cross-section, measured by the planimeter was derived as:

(2) $$S_{e}=\frac{S_{e,30}}{N_{e,30}}$$

The ear area index (EAI, one-sided projected ear area per ground unit area) was then calculated as:

(3) $$\text{EAI}=D_{e}*\;S_{e}$$

Grain yield (*R*_e_, t ha^−1^) was evaluated from the grain dry mass of the subsample ($W_{g,30}$, g) adjusted by the fraction of the dry mass subsampled and then divided by the sampled soil surface area:

(4) $$R_{e}=10\frac{W_{g,30}*B_{t}/B_{30}}{4{l*d}_{r}}$$

### 2.3 Estimation of ear density and ear surface area from phenomobile

#### 2.3.1 Acquisition of RGB images and LiDAR point cloud from the phenomobile

The AgroPhen and DiaPhen platforms are equipped with a Phenomobile V2 unmanned ground vehicle (Fig. 1c). The measurement head of the Phenomobile V2 contains LiDAR and RGB optical sensor systems. The head moves in the direction parallel to the rows at a pre-defined speed of 0.25 m s^−1^ to acquire series of RGB images and LiDAR point clouds over the microplot. Thanks to the LiDAR readings, the measurement heads is placed automatically around 1.5 m above the top of the canopy to optimize the acquisition conditions of RGB images and LiDAR point clouds.

The LiDAR system of the Phenomobile is composed of three LiDAR: two of them point towards nadir, while the third one points at 45° off-nadir (Fig. 1c). In 2022 at DiaPhen, the LiDARs were LMS400 (SICK AG, Waldkirch, Germany) with a field-of-view of 70°, a range of 3 m, an angular resolution of 0.25° along the scanning line, and a scan frequency of 300 Hz. In 2023, for both platforms, the three LiDAR were upgraded to the LMS-4124 model that has a range of 5.5 m, an angular resolution of 0.0833° along the scanning line and a frequency of 600 Hz. Thanks to a calibration protocol, the point clouds from the three LiDARs are co-registered to the same linear coordinate system. The resulting LiDAR point cloud density is about 4 and 36 millions of points per microplot for the LMS400 and the LMS-4124, respectively.

The RGB camera system of the Phenomobile V2 is composed of three Baumer VLG 40C (Baumer SAS, Fillinges, France) RGB cameras, following the same configuration as the LiDARs: two nadir-viewing cameras and a third one viewing at 45°. The cameras have a focal length of 25 mm with a field of view of 20° from the optical centre and an image resolution of 2044 × 2040 pixels. Each RGB camera acquires six images per microplots. The image acquisition is synchronized with four FR60 Xenon (Phoxene, Dardilly, France) flashes with a configurable energy level ranging from 5 to 100 J. The flashes are looking at nadir and make the image acquisition fully independent from the illumination conditions.

In each trial, three Phenomobile V2 acquisitions were conducted at three dates (Table S2) corresponding, approximately, to the growth stages (GS) anthesis (GS65), early dough (GS83), and harvest maturity (GS89) to evaluate possible differences in the accuracy linked to the colour and morphology of the ears in the segmentation of RGB images.

#### 2.3.2 Estimation of ear density using RGB and LiDAR data

**Fig. 2:**
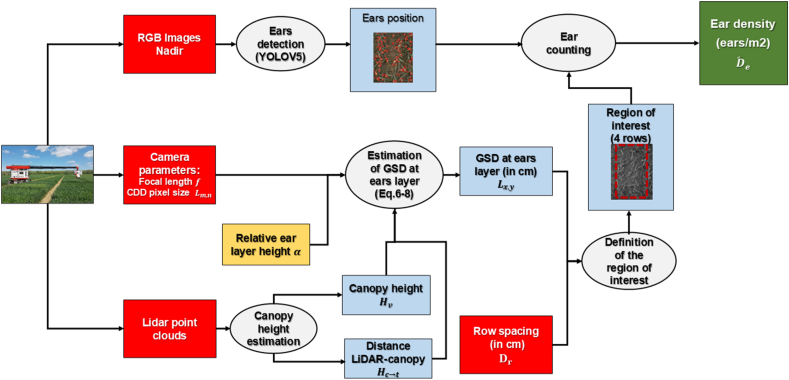
Flowchart of the method developed in this study to estimate ear density ($D_{e}$) from nadir RGB images and LiDAR point cloud obtained from the Phenomobile V2. GSD: Ground sampling distance. Red squares: input variables and datasets. Yellow square: input parameters derived from optimization. Ellipses: step of algorithm. Blue square: intermediate result.

To estimate $D_{e}$, both RGB images and LiDAR point clouds are used synergistically. The whole process is described in Fig. 2. First, ears were automatically detected in the 12 RGB images taken at nadir in each microplot by applying a deep-learning model based in the YOLOv5 CNN algorithm [23] that won the Global Wheat Head Detection Challenge (GWHDC) in 2021 and is available in https://github.com/ksnxr/GWC_solution). The algorithm achieved an overall accuracy of 0.7 across all the validation datasets in the GWHDC, see Ref. [14], and is considered a reference in the domain. The mentioned pre-trained YOLOv5 CNN model used is considered *a priori* suitable for Phenomobile V2 RGB images as they have enough resolution and are taken with active illumination, which enhances the visibility of ears in the pictures. The YOLOv5 algorithm identifies every ear in the image with a bounding box. The resulting ear density for a given microplot (${\hat{D}}_{e}$) is the median of ear density estimated on each of the 12 individual images per microplot ($D_{e,i}$ in ears/m^2^):

(5) $$D_{e,i}=\frac{N_{e,i}}{A_{ROI,i}}$$

where $N_{e,i}$ is the number of detected ears in the *i*th image, whose bounding box centroid falls inside a region of interest (ROI). The ROI is a rectangular area covering the four central wheat rows across the width of the image, and all pixels across the height of the image (see Fig. 2). The area of the ROI ${\hat{A}}_{ROI,i}$ (in m^2^) is then defined as the product of its width and height:

(6) $${\hat{A}}_{\text{ROI},i}=\left(4d_{r}\right)*\left(N_{h}*L_{x,y}\right)\cdot$$

where $d_{r}$ is the row spacing (in m), $N_{h}$ is the number of pixels across the height of the image and $L_{x,y}$ is the ground sampling distance (GSD, in m pixel^−1^) at the ears layer. GSD at the ears height is estimated as:

(7) $$L_{x,y}=\frac{H_{c\to e}\cdot L_{m,n}}{f}$$

where f is the camera focal length (in m), $L_{m,n}$ (in m) is the pixel size of the camera charge-coupled device (CCD) and $H_{c\to e}$ is the estimated distance between the camera and the ears:

(8) $$H_{c\to e}=H_{c\to t}+{\alpha \cdot H}_{v}$$

where $H_{c\to t}$ (in m) is observed distance between the camera and the top of the canopy, $H_{v}$ (in m) is the canopy height, and α (dimensionless) is a constant that expresses the proportion of the total canopy height occupied by ears. Both $H_{c\to t}$ and $H_{v}$ are derived from the Phenomobile LiDAR as explained by Ref. [6], since RGB cameras and LiDAR are placed at the same height of the measurement head. The constant α is fixed for all the trials at 0.16 (e.g. in a 1 m high canopy, ears are placed in the top 16 cm of the canopy). The value chosen α minimized the root mean squared error (RMSE) of ${\hat{D}}_{e}$ across all treatments and trials (further details are given in Section 3.1).

#### 2.3.3 Estimation of ear surface area using RGB images

**Fig. 3:**
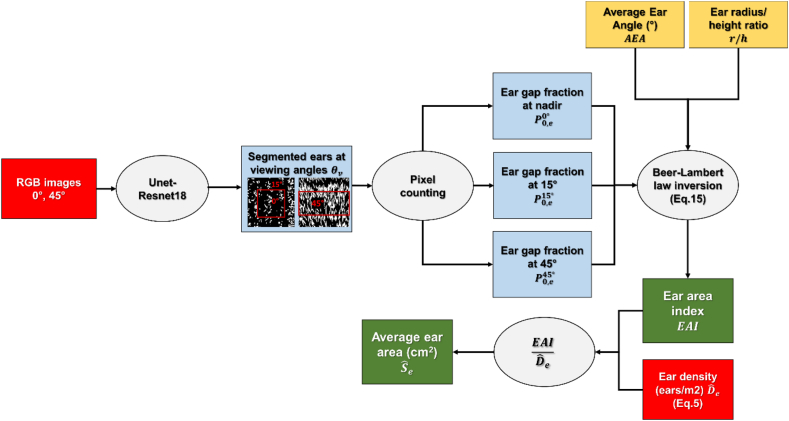
Flowchart of the method developed in this study to estimate the ear surface area and the ear area index from RGB images acquired at 0° (nadir) and at 45° from the Phenomobile V2. Red squares: input variables and datasets. Yellow squares: input parameters derived from optimization. Ellipses: step of algorithm. Blue squares: intermediate result.

The estimation of $S_{e}$ is based on the inversion of BL using the ear gap fraction $P_{0,e}$ (dimensionless) as input. The inversion of BL has been extensively applied to estimate leaf area index (LAI) in vegetation canopies from canopy gap fraction (see Ref. [22] for a review of such applications). Here, we propose an analogous approach for the estimation of the ear area index (EAI) and $S_{e}$ where some elements of the classical BL theory are adapted to be consistent with ear morphology. The workflow of the methodology followed is illustrated in Fig. 3.

The ears gap fraction $P_{0,e}$ is computed from the RGB images (nadir and 45°) acquired by the Phenomobile V2 by segmenting ears by using an Unet CNN [24] with a Resnet 18 encoder (Fig. S1).The Unet-Resnet 18 algorithm was trained using an unpublished dataset created by the Arvalis Institute (France) composed of manual annotations of 433 patches of 512 × 512 pixels extracted from RGB images acquired under passive or active illumination on different trials, varieties, locations and growth stages. The training dataset was independent from the dataset used in the present work but acquired under comparable resolution and acquisition conditions. It is important to note that in the training dataset awns were not considered in the ear masks. The accuracy of the Unet-Resnet18 to segment wheat ears was evaluated in the DiaPhen and AgroPhen trials on a random set of 44 images (22 at nadir, 22 at 45°, 20 from awned cultivars, 24 from awnless cultivars) at different growth stages, that were manually annotated.

For a given image, $P_{0,e}$ was computed as

(9) $$P_{0,e}=1-\frac{\;N_{e}}{N}$$

where $N_{e}$ (pixels) is the number of pixels segmented as ‘ear’ in the image and N (pixels) is the total number of pixels in the image. For the nadir-looking images, $P_{0,e}$ was calculated at two viewing angles: at 0° by computing $P_{0,e}$ over the pixels that are below a field of view (FOV) of 10° from the center of the image, and at 15° by computing $P_{0,e}$ over the pixels in the interval 10° < FOV <20°. For the 45° images, $P_{0,e}$ was computed selecting only the central part of the image, as illustrated in Fig. 3, to limit the FOV variability around 45°. The ear gap fraction computed at these three viewing angles are named $P_{0,e}\left(0{}^{\circ}\right)$, $P_{0,e}\left(15{}^{\circ}\right)$, and $P_{0,e}\left(45{}^{\circ}\right)$.

Then, ear area index (EAI) was estimated from $P_{0,e}$ based on an adaptation of BL to ears. We assumed that ears are represented by cylinders that are randomly distributed across the x,y plan of the image. These cylinders are assumed to be inclined following a distribution described by the ellipsoidal inclination distribution function [25], and have a random azimuth. The ellipsoidal distribution is commonly used to model leaf inclination for multiple plant species [26], and considered in this work suitable *a priori* to describe ears inclination. Furthermore, it is a model relatively simple and easy to implement that takes only a single parameter, the average angle (AEA, in °) as input. The cylinders are determined by the area of their vertical cross-section $S_{e}$ (cm^2^) and the ratio between the radius and the height of the cylinder (r/h, dimensionless). The variable $S_{e}$ corresponds to the projected ear area measured by the planimeter. BL can then be expressed as:

(10) $$P_{0,e}\left(\theta_{v}\right)=e^{-k\left(\text{AEA},\theta_{v},r/h\right)\times \text{EAI}}$$

The extinction coefficient k is given by:

(11) $$k\left(\text{AEA},\theta_{v},r/h\right)=\frac{G\left(\text{AEA},\theta_{v},r/h\right)}{\cos\left(\theta_{v}\right)}$$

where $\theta_{v}$ is the viewing angle, G refers to the gamma function that project in the direction $\theta_{v}$ a cylinder with a vertical cross-section of a unit area, a shape factor r/h, and inclined with an average angle AEA (°). The function G is given by:

(12) $$G\left(\;\text{AEA},\theta_{v},r/h\right)=\frac{1}{2\pi }\int_{\theta_{h}=0}^{\pi /2}\int_{\phi_{h}=0}^{2\pi }g\left(\theta_{h},\text{AEA}\right)\left(\pi \omega^{2}\;\cos\left(\Psi \right)+2\omega \times H\times \sin\left(\Psi \right)\right)d\theta_{h}d\phi_{h}$$

where $\theta_{h}$ and $\phi_{h}$ (°) are the zenith and azimuth angles of the ear, respectively, $g\left(\theta_{h},\text{AEA}\right)$ is the probability of $\theta_{h}$ given by the ellipsoidal inclination distribution function with AEA as input, ω (unitless) and H (unitless) are the diameter and height of the unit area cylinder, respectively, given by:

(13) $$\omega =\sqrt{\frac{r/h}{2}}$$

(14) $$H=\frac{1}{2\omega }$$

and Ψ is the phase angle between the cylinder and the viewing direction given by:

(15) $$\cos\left(\Psi \right)=\cos\left(\theta_{v}\right)*\cos\left({90-\theta }_{h}\right)+\sin\left(\theta_{v}\right)*\sin\left({90-\theta }_{h}\right)\cos\left(\varphi_{v}-\varphi_{h}\right)$$

The integral of the function G is computed numerically.

BL (Eq. (10)) can be inverted to estimate EAI by solving the following linear model using the ordinary least squares method:

(16) $$\begin{array}{c} -\ln\vec{P_{0,e}^{\theta_{v}}}=\text{EAI}\times \vec{k\left(\text{AEA},\theta_{v},r/h\right)} \end{array}$$

where $\vec{P_{0,e}^{\theta_{v}}}$ is the vector of observed ears gap fractions at different viewing angles $\theta_{v}$ and $\vec{k}$ is the known vector of the extinction coefficients for the different viewing angles. The parameter r/h was fixed at a nominal value of 0.07, representing the mean of manual measurements of the dimensions (excluding awns) of 10 ears from five of the studied varieties (Fig. S2). The angle of 65° was established as the fixed value for AEA, given the tilt of the ears during grain-filling. The effect of AEA on the accuracy of $S_{e}$ estimation and the rational for fixing AEA to 65° are both presented in the Results section.

Finally, after estimating EAI, $S_{e}$ can be derived as:

(17) $${\hat{S}}_{e}=\frac{\text{EAI}}{{\hat{D}}_{e}}$$

where ${\hat{D}}_{e}$ is the estimated value of $D_{e}$ (Eq. (5)). The algorithm to estimate $S_{e}$ from ear segmentation masks and an example dataset is accessible at https://forge.inrae.fr/raul.lopez-lozano/wheat-ear-surface.

### 2.4 Assessment of the accuracy of the algorithms proposed

#### 2.4.1 Ears segmentation algorithm

The accuracy of the ear segmentation model used to estimate $S_{e}$ was evaluated by the $F_{1}$ score [27], which measures the precision (rate of true positive predictions) and the recall (rate of correctly predicted positives).

(18) $$F_{1}=\frac{\text{TP}}{\text{TP}+\frac{\text{FN}+\text{FP}}{2}}$$

Where TP is the number of true positives (i.e. pixels segmented as ‘ear’ that were annotated as ‘ear’), FP is the number of false positives (i.e. pixels segmented as ‘ear’ that were not annotated as ‘ear’) and FN is the number of false negatives (i.e. pixels annotated as ‘ear’ that were not segmented as ‘ear). For a perfect model, $F_{1}$ equals 1 and for a non-informative model $F_{1}$ equals 2p/(p+1) where p is the rate of positives in the dataset (here the rate of pixels ‘ear’).

#### 2.4.2 Ear density and ear surface area estimation

The accuracy of the algorithms proposed for estimating $D_{e}$ and $S_{e}$ was evaluated by calculating the coefficient of determination (*r*^2^), and the root mean squared error (RMSE) between the actual ($y_{i}$) and estimated ($\hat{y_{i}}$) values, expressed in both absolute (RMSE) and relative (RMSE) values:

(19) $$r^{2}=\frac{\sum_{i=1}^{N}\left(y_{i}-\bar{y}\right)\left(\hat{y_{i}}-\bar{\hat{y}}\right)}{\sqrt{\sum_{i=1}^{N}{\left(y_{i}-\bar{y}\right)}^{2}\sum_{i=1}^{N}{\left(\hat{y_{i}}-\bar{\hat{y}}\right)}^{2}}}$$

(20) $$\text{RMSE}=\sqrt{\frac{1}{N}\sum_{i=1}^{N}{\left(y_{i}-\hat{y_{i}}\right)}^{2}}$$

(21) $$\text{rRMSE}=\frac{\text{RMSE}}{\frac{1}{N}\sum_{i=1}^{N}y_{i}}$$

where N was the number of samples.

The precision of the algorithms was also evaluated by measuring the repeatability coefficient of the predicted trait (${\text{RC}}_{\text{pred}}$) and comparing it against the repeatability coefficient of the manual observation (${\text{RC}}_{\text{obs}}$). The repeatability was computed as the root mean squared difference between the trait values measured for the two replicates of each G (genotype)/E (environment, i.e. a treatment in one location) combination:

(22) $$\text{RC}=\sqrt{\frac{1}{N_{G\times E}}\sum_{p}{\left({V_{p}}_{1}-{V_{p}}_{2}\right)}^{2}}$$

where $N_{\text{GxE}}$ is the number of cultivar/trial/treatment combination, and ${V_{p}}_{1}$ and ${V_{p}}_{2}$ are the trait values for the two replicates of the p^th^ cultivar/trial/treatment combination.

### 2.5 Statistical analysis of manual and image-based indicators across trials

An analysis of variance (ANOVA) was applied to $D_{e}$ and $S_{e}$ across the three trials to estimate the genotypic variance in relation to the total variance. The environment term ($\underline{E}$, site a year) and the management × environment interaction ($\underline{M:E}$ treatments per site) were considered fixed in the ANOVA. Management conditions correspond to the four treatments per site (sowing density or irrigation condition and sowing date). The genotypic effect (G), the interactions genotype x environment (G:E), genotype x management x environment (G:M:E) and the spatial effects nested in each environment (Row and Col) were considered as random terms:

(23) $$\begin{array}{ccc} Y\;\;\;\; & =\;\;\;\;\;\; & \underline{\mu }+\underline{E}+\;\underline{M:E}\;+G+\;G:E+\\ & & G:M:E+E:Row+E:Col+\varepsilon \end{array}$$

μ is the intercept term (fixed) and ε the random residual error. Then the Cullis heritability [28] was estimated as

(24) $$H_{cullis}^{2}=1-\frac{PEV_{g}}{2\sigma_{g}}$$

Where $\sigma_{g}$ is the genetic variance and $PEV_{g}$ is the prediction error variance associated to the genotypic effect. The analysis was done with the R statistical software, using the ‘sommer’ package [29].

Finlay-Wilkinson linear regressions [30] were computed from the results of the multi-environmental ANOVA, where the environmental index in the x-axis corresponds to centred E+M:E values and the genotype predictions are the values of the y-axis. Finlay-Wilkinson graph is used here as descriptive indicator of the genotype sensitivity to the combined environment + treatment gradient.

A second simpler ANOVA, with only genotypic and replicate (Rep) random effects, is then applied to each environment and treatment (E∗M) to estimate Cullis heritability per treatment:

(25) $$Y=\underline{\mu }+G+Rep+\varepsilon$$

The estimations of $D_{e}$ and $S_{e}$ from Phenomobile used to perform these statistical analysis are provided in the Supplemenal Materials as a separate Excel file

## 3 Results

### 3.1 Accuracy of ear density estimation from the detection of ears in RGB images and LiDAR point clouds

**Fig. 4:**
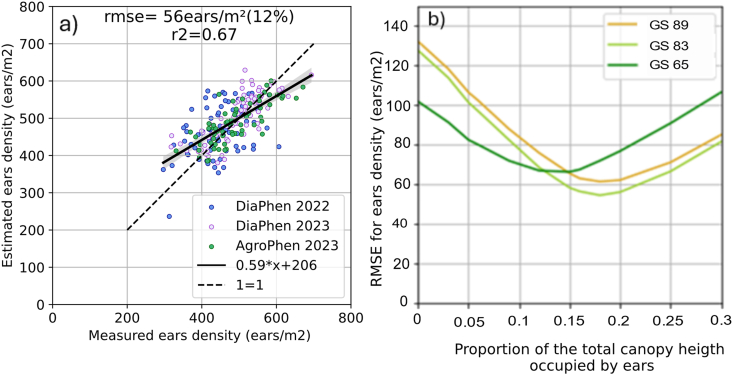
a) Ear density estimated from Phenomobile V2 observations at the growth stage GS83 versus ear density ($D_{e}$) measured destructively at harvest for 10 bread wheat varieties grown at the DiaPhen during the 2021-2022 and 2022-2023 growing seasons and at AgroPhen during the 2022-2023 growing season. Data are for individual microplots of the four treatments in each of the three trials considered in this study (n = 213 points). In total, 27 microplots were affected by lodging and not taken into account. The solid line is the linear regression across three trials, the grey area surrounding represents the 95% confidence interval. The dashed line is the 1:1 relationship. b) Root mean squared error (RMSE) for $D_{e}$ versus the proportion of the canopy height occupied by ears (parameter α in Eq. (8)). The RMSE was calculated across the 10 varieties and three trials.

The overall RMSE for $D_{e}$ calculated over the two years and the two sites was lower at GS83 than at GS65 and GS89 (Table 2). Furthermore, at GS83 $D_{e}$ estimates showed no bias for the sites and were consistently distributed along the 1:1 line (Fig. 4a) with an *r*^2^ of 0.67. The repeatability coefficient was 46 ears m^−2^ for the Phenomobile estimations (${RC}_{pred}$), against 68 ears m^−2^ for the destructive measurements (${RC}_{obs}$), indicating that $D_{e}$ estimations from Phenomobile are more consistent between the two replicates of each G x E x M combination than destructive measurements. It should be noted, however, that destructive measurements were collected over a much smaller sampling area as compared to the plot area sampled by Phenomobile. The accuracy of the estimations at GS83 in 2023 (in the two sites) is systematically higher than in 2022 (Table 2). The *r*^2^ is also systematically lower in DiaPhen 2022 (*r*^2^ < 0.5) as compared to 2023 (0.61 < *r*^2^ < 0.81).

**Table 2:** Root mean squared error (RMSE), relative RMSE (rRMSE) and coefficient of determination (r2) for ear density ($D_{e}$) depending on the phenological stage at the date of observation, the sowing treatment and the trial. For each row, the phenological stage which has the lowest RMSE or rMRSE or the highest r2 value is highlighted in bold font and underlined.

Trial	Sowing treatment	RMSE (ears m^−2^)			rRMSE (%)			*r*^2^		
		GS65	GS83	GS89	GS65	GS83	GS89	GS65	GS83	GS89
*DiaPhen 2022*	S1	75	**73**	84	18	**16**	18	0.22	**0.46**	0.35
*DiaPhen 2022*	S2	74	**70**	77	17	**16**	17	**0.58**	0.48	0.46
*DiaPhen 2023*	S1	**37**	42	52	**7**	8	10	**0.7**	0.61	0.55
*DiaPhen 2023*	S2	101	**49**	50	22	**11**	11	0.57	0.68	**0.74**
*AgroPhen 2023*	S1	47	**45**	52	10	**9**	11	0.79	**0.81**	0.74
*AgroPhen 2023*	S2	55	**45**	50	11	**9**	10	0.67	**0.72**	0.66

*Overall*		68	**56**	63	14	**12**	13	0.66	**0.67**	0.61

The parameter α in (Eq. *8*), necessary to compute the image ground sampling distance at the ear level, has a strong effect on $D_{e}$ estimation (Fig. 4b). For each of the three phenological stages, the RMSE reached a minimum value of α between 0.1 and 0.2 of the canopy height. The value of 0.16 set for α, which means that ears occupy the upper 16% of the canopy height, was selected from the analysis of the results shown in Fig. 4b, and constitutes a nominal value suitable for all the three studied stages.

### 3.2 Accuracy of ear surface area estimation from ear segmentation and Beer-Lambert law inversion

Our ear segmentation model provided accurate results when evaluated over an independent dataset of 44 manually annotated images of size 512∗ 512 pixels: F1 score (Eq. (1)*8*) was 0.88 for the RGB images taken at 45° and 0.89 for those taken at nadir. The differences in the F1 score were subtle between awned (0.84 for 45° images, 0.87 for nadir images) and awnless cultivars (0.9 for both nadir and 45° images). Following the results presented above for $D_{e}$, its estimates obtained at GS83 were used to calculate $S_{e}$ from EAI (Eq. (1)*7*). The accuracy metrics for $S_{e}$ estimated at different growth stages for the three trials are given in Table 3. The error between measured and estimated $S_{e}$ is significantly higher for awned varieties compared to awnless ones. The overall RMSE for $S_{e}$ over the three trials was 3.65 (41%) and 1.31 (18%) cm^2^ for awned and awnless varieties. The accuracy of $S_{e}$ estimations –especially for awnless varieties– is slightly higher at GS83 than at GS89, while the error at GS65 is substantially higher than at the other two growth stages.

**Table 3:** Root mean squared error (RMSE), relative RMSE (rRMSE) and coefficient of determination (r^2^) for ear surface area ($S_{e})$ depending on the phenological stage at the date of observation, the sowing date, the trial and the awnedness of the varieties. For each row, the phenological stage which has the lowest RMSE or rMRSE, or the highest r2 value is highlighted in bold font and underlined.

Trial	Sowing date treatment	awns	RMSE (cm^2^)			rRMSE (%)			*r*^2^		
			GS65	GS83	GS89	GS65	GS83	GS89	GS65	GS83	GS89
DiaPhen 2022	S1	awned	4.8	**2.2**	2.3	50	**31**	31	0.1	−0.18	**0.29**
		awnless	**0.3**	1.4	1.6	**5**	24	27	−0.81	**0.62**	0.55
	S2	awned	3.6	**3.2**	3.8	52	**44**	54	0.61	0.2	**0.53**
		awnless	1.6	**0.7**	1.1	29	**13**	19	0.54	**0.77**	0.68
DiaPhen 2023	S1	awned	6.7	4.5	**4.4**	62	51	**51**	0.27	−0.22	**0.14**
		awnless	*2.3*	**0.9**	1.4	32	**14**	21	0.56	**0.4**	0.36
	S2	awned	7.8	5.7	**5.4**	84	61	**58**	**0.35**	−0.2	0.27
		awnless	4.1	2.1	**1.6**	62	31	**23**	**0.65**	0.43	0.5
AgroPhen 2023	S1	awned	3.9	1.2	**0.9**	41	14	**10**	0.19	**0.63**	0.51
		awnless	2.3	**1.0**	1.5	30	**14**	21	0.48	0.77	**0.78**
	S2	awned	3.0	1.6	**1.5**	34	18	**17**	0.11	**0.45**	0.39
		awnless	3.1	**0.8**	1.5	44	**11**	21	0.05	**0.51**	0.46
Overall		awned	5.5	3.7	**3.6**	61	41	**41**	−0.18	−0.15	**0.06**
		awnless	2.9	**1.3**	1.5	39	**18**	20	0.38	0.57	**0.66**

**Fig. 5:**
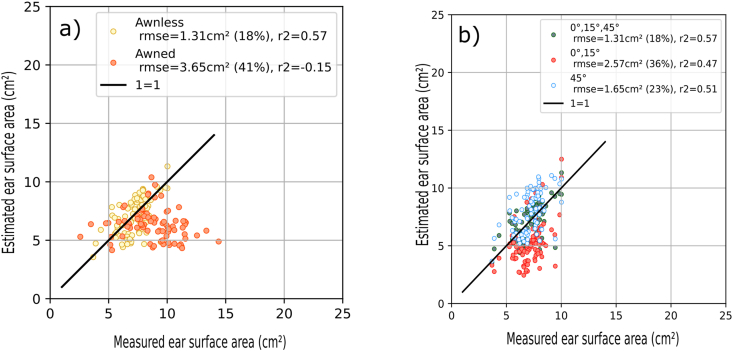
Ear surface area ($S_{e}$) estimated from Phenomobile V2 RGB images at growth stage GS83 versus $S_{e}$ measured from destructive sampling at harvest. (a) Yellow and red symbols show awnless and awned varieties, respectively. (b) $S_{e}$ was estimated from Phenomobile V2 RGB images at 0°, 15°, and 45° (light red circles), at 0° and 15° (red symbols), or at 45° (blue symbols). The average ear angle was set at 65° to estimate $S_{e}$ using (Eq. (16)). Solid lines are 1:1 relationship. Data are for individual plots of the four treatments in the AgroPhen 2023 and DiaPhen 2023 trials considered in this study.

The estimations of the $S_{e}$ at GS83 for awnless varieties are well distributed along the 1:1 line ($r^{2}$ = 0.57; Fig. 5a). In contrast, a systematic underestimation of $S_{e}$ is observed for the awned varieties in a relatively large set of observations. Specifically, $S_{e}$ measured in the laboratory with the LiCOR-3100 instrument can be two or even three times higher than estimated $S_{e}$ from the Phenomobile. These discrepancies between measured and estimated $S_{e}$ for awned varieties are mainly coming from the DiaPhen trials. For awned varieties the rRMSE are higher at DiaPhen (38% and 56% considering the two sowing dates in 2022 and 2023, respectively) than at AgroPhen (18% in 2023). The repeatability coefficient of the Phenomobile estimations (0.53 cm^2^) is half that of the manual measurements (1.05 cm^2^). This result indicates that Phenomobile-based estimations are more coherent than manual measurements across replicates of the same G x E x M combinations.

**Fig. 6:**
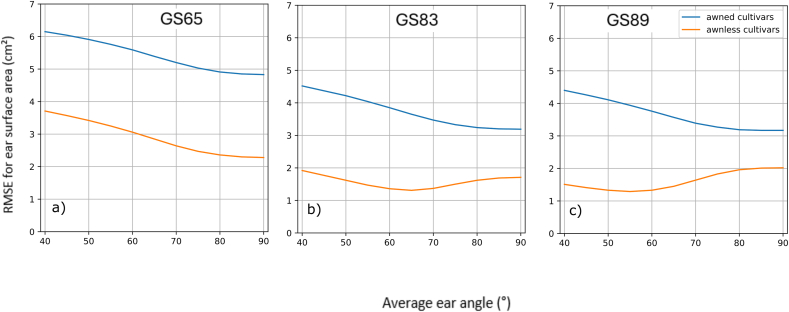
Root mean squared error (RMSE) for $S_{e}$ for awned (blue lines) and awnless (orange lines) varieties as a function of the average ear angle (AEA) used to invert the Beer-Lambert law (see section 2.3.3) at growth (a) anthesis (GS65), (b) early dough (83), and (c) maturity (GS89).

If we focus on awnless varieties, two factors determine the accuracy of $S_{e}$ estimation. The first factor is the viewing geometry of RGB images (Fig. 5b). The best performance is achieved when BL is inverted with gap fraction at 45°, either alone (RMSE = 1.65 cm^2^) or combined with 0° and 15° gap fraction from nadir-looking RGB images (RMSE = 1.3 cm^2^). The accuracy degrades substantially when BL is inverted using only gap fraction from nadir-looking RGB images (RMSE = 2.57 cm^2^). The second factor is the value chosen for AEA when inverting BL in (Eq. (1)*6*). Fig. 6 plots the variability of the error between measured and estimated $S_{e}$ depending on the value for AEA chosen. For awnless varieties the value of AEA that minimizes the error depends on the growth stage of observation (Fig. 6). It is 90°, 65°, and 55° at GS65, GS83, and GS89, respectively. These results indicate that the optimal value for AEA decreases progressively as the phenological stage gets close to maturity, which agrees with the observable fact in the field that the erectness of ears decreases from the heading stage onwards. The nominal value of AEA of 65° used for BL inversion corresponds to the optimal value at the GS83 stage. Nevertheless, a comparable accuracy could be obtained at GS89 by fixing the AEA to 55° (RMSE = 1.3 cm^2^ for awnless varieties; Fig. 6c). The best performance obtained at GS65 when considering that ears are erect (AEA = 90°, RMSE = 2.3 cm^2^ for awnless varieties; Fig. 6a), is far from the accuracies achieved at GS83 and GS89. For awned varieties, AEA = 90° yields the lowest RMSE for $S_{e}$ at the three phenological stages (Fig. 6). However, as mentioned earlier, in this group of varieties there are large discrepancies between measured and observed surface area (RMSE ranging between 3 and 5 cm^2^ depending on the growth stage) as compared to the awnless group.

### 3.3 Heritability of ear density and ear surface area estimations from phenomobile

**Fig. 7:**
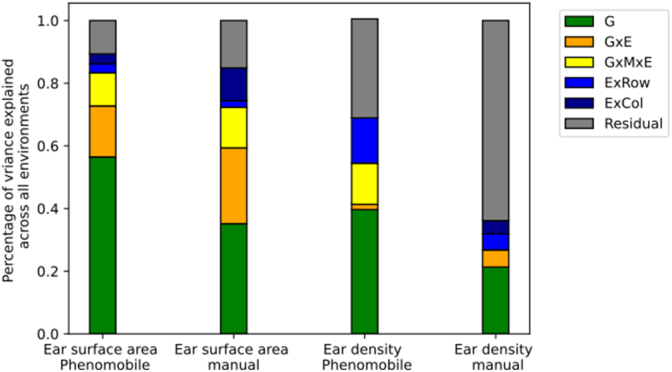
Percentage of variance of ear density ($D_{e}$) and ear surface area ($S_{e}$) estimated from Phenomobile V2 data and measured across all environments explained by different factors. The following factors were considered in the ANOVA: row, column, genotype, genotype by environments, genotype by environment and treatment and the residual. Management conditions is defined by sowing date x sowing density (only at AgroPhen) and water treatment x sowing date (only at DiaPhen).

The genotype is the main factor explaining the variance of both, ear density and ear surface area estimations from Phenomobile across all environments, as shown in Fig. 7. The proportion of variance explained by Phenomobile $S_{e}$ and $D_{e}$ is estimated at 56% and 40%, respectively in the multi-environment model described in (Eq. (2)*3*). The genotype explains a higher proportion of variance when both traits are estimated by Phenomobile as compared to manual measurements. The contribution of residual errors to total variance for Phenomobile measurements are systematically lower than those observed for manual measurements, especially for manual $D_{e}$, where residuals represent more than 60% of total variance (Fig. 7).

The Cullis heritability across environments is high for both manual and Phenomobile observations of ear density and ear surface area ($H_{cullis}^{2}$ > 0.85). Nevertheless, the retrieved $H_{cullis}^{2}$ for Phenomobile estimations are higher (0.92 for $D_{e}$, 0.90 for $S_{e}$) than manual observations (0.87 for $D_{e}$, 0.85 for $S_{e}$). Similarly, the heritability calculated separately per environment and treatment (Table 4) indicates as well that, in practically all the cases, the $H_{cullis}^{2}$ of Phenomobile estimations of $D_{e}$ is substantially higher than manual measurements. For $S_{e}$ the heritability values are closer between Phenomobile estimations and manual measurements, with perhaps the exception of the AgroPhen 2023 trial.

**Table 4:** Cullis heritability for ear surface area ($S_{e})$ and ear density ($D_{e}$) measured and estimated from Phenomobile V2 data, by environment and management conditions. The genotype and the repetition were considered as factors in the ANOVA. The underlined values indicate the method with the highest heritability for each trait and management factor.

		Ear density		Ear surface area	
Trial	Management	Phenomobile	Manual	Phenomobile	Manual
**DiaPhen_2022**	WWS1	0.76	**0.82**	0.9	NA
	WDS1	0.83	**0.85**	0.95	NA
	WWS2	**0.90**	0∗	0.81	NA
	WDS2	**0.87**	0.74	0.93	NA
**DiaPhen_2023**	WWS1	**0.77**	0∗	**0.97**	0.95
	WDS1	**0.93**	0.75	0.94	**0.96**
	WWS2	**0.95**	0.57	0.95	**0.96**
	WDS2	**0.87**	0.57	**0.95**	**0.95**
**AgroPhen_2023**	D200S1	**0.83**	0.74	**0.97**	0.77
	D200S2	**0.87**	0.79	**0.96**	0.82
	D400S1	**0.81**	0.78	**1.0**	0.93
	D400S2	**0.89**	0.63	**0.96**	0.77

∗genotypic variance was estimated as zero, indicating no detectable genetic differences under these conditions.

### 3.4 Cultivar and environmental variability of ear density and ear surface area estimated from phenomobile

**Fig. 8:**
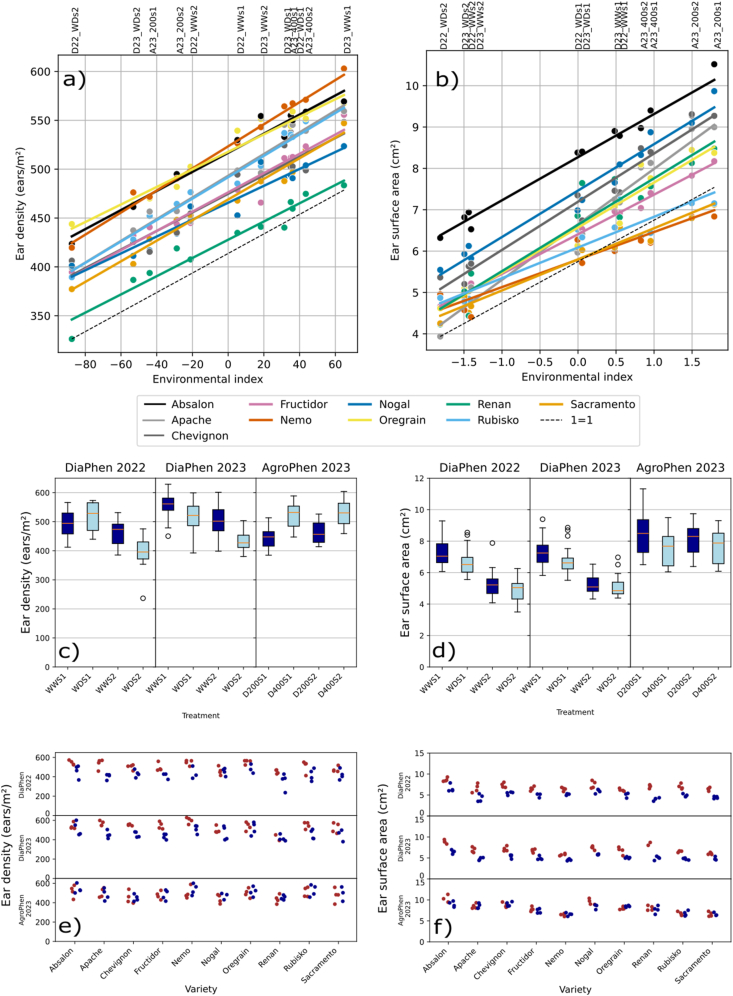
Finlay-Wilkinson regression for Phenomobile $D_{e}$ (a) and $S_{e}$ (b) constructed from the multi-environment ANOVA (Eq. (23)).Boxplot of ear density ($D_{e}$, c) and ear surface area ($S_{e}$, d) estimated from the Phenomobile V2 images grouped by sowing and treatments for DiaPhen 2022, DiaPhen 2023 and AgroPhen 2023 trials. Jitter plots of $D_{e}$ (e) and $S_{e}$ (f) estimated from Phenomobile per variety and sowing date in the three trials considered in this study. Red dots in the jitter correspond autumn sowings and blue dots correspond winter sowings. Treatments: S1, autumn sowing; S2, winter sowing; WW, irrigated; WD, rainfed; D200, 200 seeds m-2, D400, 400 seeds m-2.

Fig. 8a displays the Finlay-Wilkinson graphs for $D_{e}$ computed after the multi-environment mixed model presented in Section 2.5. Important varietal differences are observed across sites and treatments: the analysis highlights a group of three genotypes (Absalon, Nemo and Oregrain) where $D_{e}$ is systematically higher as compared to the other genotypes in almost all the environments, and Renan, on the opposite situation, presents a low ear density. Cultivar rankings across treatments and environments are coherent, with Nemo, Absalon and Oregrain consistently in the first positions.

The regression slopes in Fig. 8a show that Apache, Nemo and Rubisko present a slightly higher responsiveness in $D_{e}$ across the sites and treatment. The main differences between treatments are coming from autumn and winter sowings of these three varieties in the two DiaPhen trials (Fig. 8e) where $D_{e}$ is systematically higher in autumn sowings (S1) than in winter sowings (S2) in both years. This influence of the sowing date in $D_{e}$ is observed in most genotypes, and is more evident in the rainfed (WD) than in the irrigated (WW) treatment (Fig. 8c). Absalon, Oregrain and especially Nogal (a cultivar with low cold requirements) are those presenting the smallest differences in $D_{e}$ between winter and autumn sowings (Fig. 8a–e).

In the AgroPhen 2023 trial the differences in $D_{e}$ are driven by the initial sowing density –$D_{e}$ 20% higher for the 400 seeds m^−2^ treatment as compared to 200 seeds m^−2^– and only a marginal influence of the sowing date is observed (Fig. 8c). Rubisko and Apache exhibit a more pronounced response than the other genotypes to an increase in the sowing density in AgroPhen. It should be noted that the difference in days between the autumn and winter sowing dates in the AgroPhen 2023 trial is small as compared to DiaPhen (29 days in AgroPhen 2023, 56 and 71 in DiaPhen 2022 and DiaPhen 2023, Table 1).

Regarding $S_{e}$, Absalon, Nogal and Chevignon are identified as the three varieties with the largest $S_{e}$ and Nemo, Rubisko, and Sacramento are clearly those with the smallest $S_{e}$ (Fig. 8b). Nevertheless, the slope of the Finlay-Wilkinson regression reveals a rather different response of the cultivars to the different treatments. The cultivars Absalon, Renan and Chevignon –the three with a high average $S_{e}$– and Apache show the most pronounced response of $S_{e}$ to the treatments and trials. Particularly Apache shows the lowest $S_{e}$ in the less favourable environments but ranks 4th in the most favourable ones. By contrast Nemo, Rubisko and Sacramento –the three cultivars with the smallest ears– present the lowest responsiveness, and correspond to those cultivars who exhibited a larger $D_{e}$ response to the environment.

As it can be observed in Fig. 8b and d, the main factor influencing $S_{e}$ is the sowing date, particularly in the DiaPhen trials, which is about 50% higher for autumn than for winter sowings in most of the varieties. According to manual measurements at harvest, the changes in ear length between autumn and winter sowings for all varieties are rather small (about 0.25-0.5 cm, see Fig. S3), which indicates that these large $S_{e}$ differences introduced by the sowing date are mainly given by ear width. The highest $S_{e}$ are observed in the four treatments of the AgroPhen trial (Fig. 8b–d), where the effect of management in $S_{e}$ appears to be minimal and the within-genotype variance is low (Fig. 8f). Interestingly, a slight compensation effect between $S_{e}$ and $D_{e\;}$ can be appreciated in the sowing density treatments at AgroPhen (Fig. 8c and d).

### 3.5 Relationship between estimated ear density, ear surface area and yield components

**Fig. 9:**
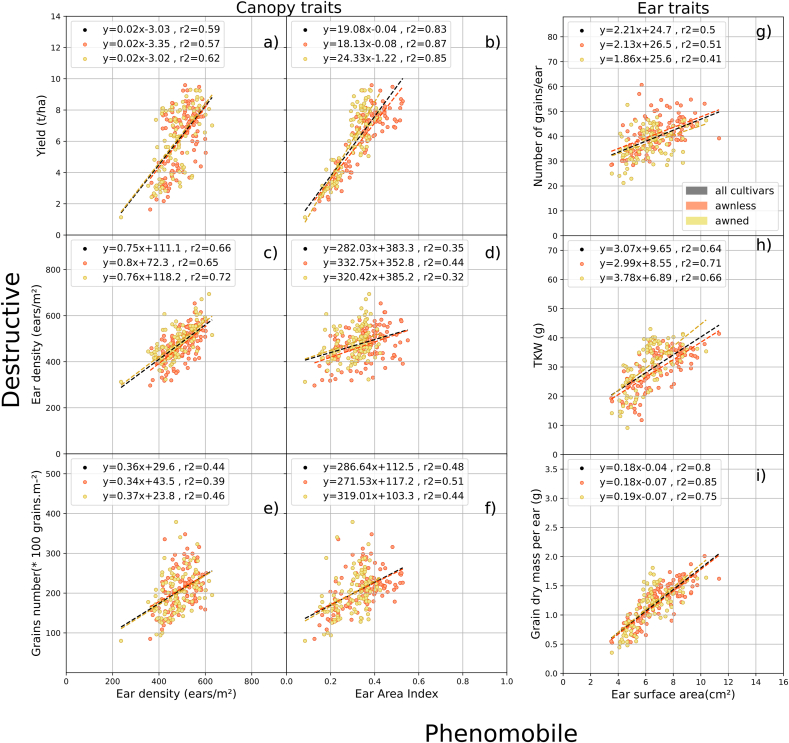
Relationship between yield components and traits estimated from the Phenomobile V2 at growth stage GS83. (a – f) Grain yield (a, b), ear density (c, d) or grains number per m2 (e, f) versus ear density (a, c, d) or ear area index (EAI; b, d, f). (g – i) Grain dry mass per ear (i), number of grains per ear (g) and thousand kernels weight (h) versus ear surface area. Data are the values measured or estimated for single microplots (n = 213). In total, 27 microplots were affected by lodging and not taken into account. Lines are linear regression (all P < 0.01) fitted to the awnless (red lines), awned (yellow lines), and all varieties (black lines).

Fig. 9 shows the relationship between different yield components measured destructively and the ear traits estimated from Phenomobile at the canopy scale ($D_{e}$ and EAI) and at the ear scale ($S_{e}$).

At the canopy scale, the estimated EAI from Phenomobile is strongly and positively correlated with grain yield ($r^{2}=$ 0.83, *P* < 0.01; Fig. 9b). Although the relationship between $D_{e}$ estimated from Phenomobile and grain yield is also statistically significant ($r^{2}=$ 0.59, *P* < 0.01; Fig. 9a), the coefficient of determination is substantially lower compared to EAI. Both EAI and $D_{e}$ estimated from Phenomobile are also correlated –but rather weakly – with the number of grains per m^2^ ($r^{2}<$ 0.50, *P* < 0.01; Fig. 9e and f).

At the ear-level, the estimated $S_{e}$ from Phenomobile exhibits a strong and positive correlation with grain dry mass per ear ($r^{2}=$ 0.8, *P* < 0.01 Fig. 9i). A closer look at the relationship between $S_{e}$ and the two yield components that determine grain dry mass per ear shows that $S_{e}$ is better correlated with TKW ($r^{2}=$ 0.64, *P* < 0.01 Fig. 9h) than with the number of grains per ear ($r^{2}=$ 0.5, *P* < 0.01 Fig. 9g). Indeed, $S_{e}$ is also directly correlated with yield ($r^{2}=$ 0.69, *P* < 0.01, not shown in Fig. 9), but this is explained because TKW, and not ear density, is the main component determining yield across the trials (Table S3).

**Fig. 10:**
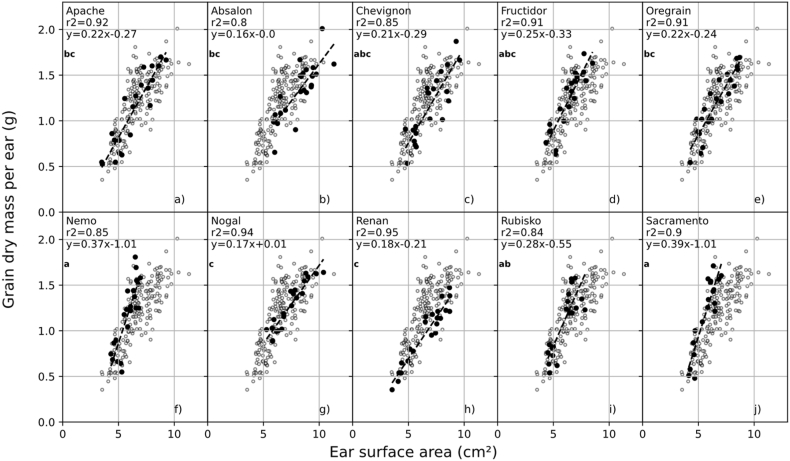
Relationship between grain dry mass per ear and estimated ear surface area from the Phenomobile V2 for the 10 variety used in this study (black circles). (a – e) Awnless varieties. (f – j) Awned varieties. Data are the values measured or estimated for single microplots in the three trials considered in this study (n = 213). In total, 27 microplots were affected by lodging and not taken into account. Lines are linear regression (all P < 0.01) fitted to each variety. The empty circles represent data for all 10 varieties shown to facilitate the visual comparison of the varieties. Letters in bold indicate statistically significant differences in the slope between varieties at 95% confidence interval.

The relationship between the estimated $S_{e}$ and the grain dry mass per ear is variety-specific (Fig. 10), with $r^{2}$ ranging between 0.80 for Absalon and 0.95 for Renan. The variability in the slope of the linear models between varieties is statistically significant for some groups of varieties. Sacramento exhibits the steepest slope (0.39 ± 0.05 g cm^−2^; Fig. 10j), closely followed by Nemo (0.37 ± 0.05 g cm^−2^; Fig. 10f), meaning that they require a low increment in $S_{e}$ to achieve a larger amount of grain dry mass per ear. Indeed, these two varieties are, systematically, the ones with the shortest ears across all the environments (see Fig. S3), and those presenting the lowest responsiveness of $S_{e}$ to the environment (Fig. 8b). At the other end of the spectrum, Absalon*,* Nogal and Renan, which have the highest $S_{e}$ across all the environments, have smaller slopes (ranging from 0.16 ± 0.03 g cm^−2^ to 0.18 ± 0.01 g cm^−2^; Fig. 10b–g,h). Absalon and Renan are the two varieties with the longest ears (see Fig. S3). Similarly, differences in the slope of the correlation between EAI and grain yield are statistically different between some varieties (Fig. S4). The range of grain dry mass per ear for most varieties is relatively broad, spanning from 0.5 to 1.8 g ear^−1^. For some varieties (e.g. Sacramento, Renan, and Rubisko) the grain dry mass per ear are clearly distributed around two groups, corresponding to two contrasted yielding environments: a first one centred around 0.6 to 0.7 g corresponding to the late winter sowings (S2) at DiaPhen in 2022 and 2023 (both rainfed and irrigated); and a second one around 1.4 to 1.5 g, corresponding to the remaining high-yielding environments. By contrast, for other varieties such as Apache, Fructidor, and Nogal, the observed grain dry mass per ear is more uniformly distributed across the environments. Nogal –a spring type variety – is the one presenting the lowest variance across environments. These environmental effects on grain dry mass per ear are well described by the estimated $S_{e}$ from Phenomobile. $S_{e}$ is also strongly correlated with TKW for all varieties (Fig. S5), while for most varieties it is more weakly correlated with the number of grains per ear (Fig. S6).

It should be noted that the correlations observed between grain yield or its components and $S_{e}$ or EAI measured manually are much weaker (Fig. S7) than those for $S_{e}$ and EAI estimated from the Phenomobile. The weaker correlations of manual measurements are caused by awned varieties, for which large discrepancies were observed between destructive measurements estimations from Phenomobile of $S_{e}$ (Fig. 5).

## 4 Discussion

### 4.1 Reliability of the proposed methods to estimate ear density and ear surface area for plant phenotyping

In the current study we have presented a method combining RGB and LiDAR data to estimate wheat $D_{e}$ at the microplot scale. The achieved RMSE of 56 ears m^−2^ (12% rRMSE) is slightly lower than that documented by Ref. [31] who reported an RMSE of 71.4 ears m^−2^ (14% rRMSE) and [12] who reported an RMSE of 69 ears m^−2^ (∼15% rRMSE). Compared to these previous studies, three factors may explain the enhanced performance achieved in the present study. Firstly, the controlled light environment during Phenomobile acquisitions avoids the effects of shadows projected over some ears that may cause a systematic under detection when working under natural light, as indicated by Ref. [11]. Secondly, the LiDAR in the measurement head of the Phenomobile allows to estimate the distance between the camera and the top of the canopy. This information is essential to determine accurately the area observed in the RGB images and, therefore, to select a sampling area equivalent to a multiple of the row spacing to avoid any possible bias. Also [12] highlighted the importance of a robust approach to determine the sampling area to mitigate possible bias in the estimated $D_{e}$. The third factor is the use of YOLOV5 CNN –the best model of the GWHD challenge, see Ref. [14]– to detect the wheat ears. This CNN was trained on a global dataset of wheat images, which should *a priori* improve the robustness of ear detection as compared to other deep neural networks trained exclusively with local datasets.

The proposed approach for estimating $D_{e}$ from Phenomobile has also demonstrated a repeatability 48% higher in comparison with manual ear counting at harvest, and also a higher heritability across environments (0.92 versus 0.87) and per environment and treatment (see Table 4). Furthermore, the RMSE of the Phenomobile estimations decreased drastically between 2022 and 2023 (Table 2). This was because the ears were counted manually in 2023 on a subsample of 30% of the collected dry mass at harvest against 15% in 2022. This suggest that manual ears counting may suffer from important uncertainties associated to the representativeness of the sampled area, whereas automatic methods based on RGB imagery can easily overcome this problem by taking enough images per plot, especially in trials as the current one where plot size is large. This hypothesis about the representativeness of manual measurements is reinforced by the results of the variance decomposition analysis. For Phenomobile estimations the residual explains 30% of the variance across environments (Fig. 7) whereas for manual measurements residual represented up to 60%. Considering all these evidences, we believe that traditional manual ear counting can be safely replaced in wheat phenotyping trials by CNN-based methods as the one proposed in this study.

Thanks to the estimation of $D_{e}$, we have developed an original approach to estimate $S_{e}$ relying on the computation of the ear gap fraction and EAI from the automatic segmentation of RGB images. Although several ear segmentation models have been proposed with promising results [11,32,33] to the best of our knowledge only Zhang et al. [21] have attempted to estimate ear dimensions, reporting a relative mean absolute error of 24% in volume estimations as compared to ear dimensions derived from LiDAR point-clouds. Our results indicate a satisfactory agreement between manual measurements of $S_{e}$ and Phenomobile estimations for awnless varieties (18% rRMSE) but large discrepancies for awned varieties (41% rRMSE). These large discrepancies observed in awned varieties are due to the impact of awns in the projected ear area measured by the LiCOR-3100 instrument, observed particularly in the DiaPhen trial. The presence of awns can produce inconsistent measurements of the ear area across varieties and treatments since awns may be systematically over-detected or under-detected by the LiCOR-3100 instrument depending on the awns diameter and length and the position of the ear in the instrument (see outliers in Fig. 5a). These inconsistencies for awned varieties probably increased artificially the heritability in the treatments of the DiaPhen trial (Table 4), where the differences in the ear surface area measured by the LiCOR-3100 instrument for awned and awnless cultivars were large.

Furthermore, when considering $S_{e}$ as a trait possibly related to the number of grains per ear or to grain dry mass per ear, accounting for the awn surface area is probably a disadvantage. Actually, the correlation between manual $S_{e}$ and grain dry mass per ear was not statistically significant in awned varieties ($r^{2}$ = 0.18, *P* = 0.06; Fig. 6) whereas for Phenomobile estimates of $S_{e}$ the correlation with grain dry mass per ear was strong ($r^{2}$ = 0.74, *P* < 0.01; Fig. 9e). Indeed, the strong correlations between the estimated $S_{e}$ from Phenomobile and the grain dry mass per ear and other yield components also proves indirectly the reliability of the proposed methodology to estimate the actual value of $S_{e}$. The automatic segmentation of wheat ears in RGB images (here conducted with the Unet-Resnet18 algorithm) constitutes, according to our results, the most efficient method to achieve a consistent estimation of $S_{e}$ as it removes the contribution of awns. Of course, measurements of ear length and height with a ruler or a calliper would be highly reliable and would also exclude awns, but at the cost of decreasing dramatically the throughput when sampling a sufficiently large number or ears per plot.

### 4.2 Generalization of the proposed algorithms to other phenotyping devices and trials

We consider that the algorithms proposed in this study to estimate $D_{e}$ and ears surface area can be easily applied to other similar devices equipped to acquire RGB images at nadir and 45°. The YOLOv5 algorithm used to detect and count ears [14] is publicly available and trained with a global dataset of RGB images taken with multiple cameras, illumination conditions and viewing geometry, so it should provide *a priori* reliable ear counting from images taken by different instruments. Although we relied on a non-public model for ear segmentation to estimate $D_{e}$, the global wheat full semantic segmentation dataset (GWFSS, [34]) provides a comparable large-scale dataset designed to train robust and accurate models for ear segmentation. Moreover, new advanced deep-learning approach, such as SAM3 [35] provides grounds for increasing the generalization of detection and segmentation of ears across a wide range of contexts. More important is the method to derive the image sampling area, needed to compute $D_{e}$. Here we benefited from the LiDAR system of the Phenomobile to compute the distance between the RGB camera and the top of the canopy, but other instruments may have only RGB cameras. Dandrifosse et al. [11] used point clouds from RGB camera stereovision as a valid alternative to LiDAR point clouds. Another option is to place a sampling square with known area over the canopy (e.g. in Ref. [31]) but this method is less efficient for automatic data collection.

The algorithm for $D_{e}$ relies on the parameter α to derive the ear sampling area from the distance between the camera and the top of the canopy. In their work Dandrifosse et al. [11] used a somewhat equivalent variable describing the ear layer height (set at 5 cm), while Li et al. [32] considered a fixed height of 11 cm for the ear layer in UAV images. In our method, rather than an absolute variable we considered a relative parameter to the height of the top of the canopy, assuming that there may be an allometric relationship between the length of the stem and the length of the ear [16]. We evaluated α across different environments and genotypes resulting in a relatively large variability of final plant height (from about 70 cm to 105 cm). Consequently, we consider α to be an effective parameter and believe that 0.16 –as used in this study– is a suitable value *a priori*, regardless of the instrument used to measure the distance between the camera and the top of the canopy. Of course, it can also be easily derived in Eq. (8) by minimising the bias against ground truth measurement, as in this study.

Images at taken at 45° are necessary (alone or jointly with nadir-viewing images) to achieve the best estimation of $S_{e}$ (Fig. 5). The accuracy of the proposed algorithm depends on parameters AEA and r/h. In this study, r/h was set at 0.07 after manually measuring the dimensions of ears of different varieties, although the results show some differences between varieties (Fig. S2). Additionally, the differences in $S_{e}$ across environments are related to the width of the ears –since ear length does not vary substantially, see Fig. S3– thus altering the parameter r/h even for a given variety. Nevertheless, we consider that fixing it to a nominal value of 0.07 will not produce *a priori* a major impact in EAI or $S_{e}$, especially when using images at 45° because r/h determines the projected area of the top side of the ear, which has only a substantial influence on EAI in nadir-viewing images when ears are perfectly vertical (AEA ∼ 90°).

On the other hand, choosing an appropriate value for AEA is much more important than r/h. Our results on awnless varieties (Fig. 6) indicate that the appropriate value for AEA depends on the development stage, decreasing progressively from flowering (optimal AEA = 90°) to physiological maturity (optimal AEA = 55°). At GS83 and GS89 using the optimal value for AEA (65° and 55°, respectively) will lead to the same accuracy. The RMSE does not change substantially within an interval of about ±10° around the optimal value of AEA (Fig. 6), which suggests that setting it at 55° for images taken around GS89 (a stage easy to identify *de visu*) is safe enough to achieve satisfactory results for other phenotyping instruments with a camera configuration similar to that of the Phenomobile. As demonstrated by Ref. [36,37] for leaf area index estimation, when inverting BL at viewing angles far from nadir, the projection function in BL (G function in Eq. (11)) is less dependent or even independent (i.e. at viewing angle of 57°) from inclination. Therefore, the use of images at 45° to compute the ears gap fraction makes the estimation of EAI and $S_{e}$ less influenced by AEA as compared to a configuration using only images taken from nadir. Considering that measuring AEA in the field is very challenging. Retrieving it by optimization at a given growth stage across genotypes and environments, as in this study, is probably the most suitable way to find an appropriate value. The optimal value chosen seems to be robust, even when derived from a reduced dataset (Fig. S8) but of course, there may be differences in AEA between genotypes at GS83 and GS89 that are neglected by our method which assumes a fixed value, and this could introduce some differences in the estimated values of $S_{e}$, and perhaps explain some of the differences in the variety-specific relationships between $S_{e}$ and grain dry mass presented in Fig. 10. More sophisticated ways to determine explicitly AEA from point clouds (e.g. LiDAR, stereo-vision) can be considered, but they require the three-dimensional segmentation of detailed point clouds, which was out of the scope of this study.

### 4.3 Ear density and ear surface area can help analyse genotype by environment interactions for yield components

The high-throughput estimation of $D_{e}$ and $S_{e}$ can help to analyse G × E interactions. At DiaPhen, autumn sowings led to a systematically higher $D_{e}$ as compared to winter sowings, and these differences are more noticeable in the rainfed treatments where late sowings are more prone to suffer from water deficit during the early reproductive phase thus impacting the number of fertile tillers per plant [38,39]. $S_{e}$ varies much more with the sowing date than with the water treatment (Fig. 8d). January sowings resulted in smaller ears as compared to November sowings, especially for varieties like Renan (a very late variety) or Absalon with differences by a factor of 2. As indicated by previous studies the length of the stem elongation phase determines $S_{e}$ [40,41]. Therefore, the shortening of that phase in late sowings in the DiaPhen trials is likely the main cause of such differences in $S_{e}$. In comparison, the influence of the sowing date on $D_{e}$ and $S_{e}$ in the AgroPhen trial was minimal, since the number of days between the two sowing dates was much lower than that at DiaPhen (Table 1). Actually in a high-yielding environment such as in the AgroPhen trial, the genotypic differences in $S_{e}$ can be clearly identified. It is important to highlight that the observed variability in $S_{e}$ between varieties at AgroPhen is coherent with that observed for the autumn sowings at DiaPhen (Fig. 8b–f, winter sowing series), which demonstrates that $S_{e}$ estimations from Phenomobile are robust across trials.

$S_{e}$ and EAI are strongly correlated with the grain dry mass per ear (*r*^2^ = 0.8 across varieties) and the grain yield (*r*^2^ = 0.83), respectively (Fig. 9), which suggests that an indirect, automatic method determining $S_{e}$ (and hence EAI) can provide proxies for grain yield and important yield components. The correlation between EAI and grain yield is also statistically significant at GS65 (Fig. S9), which indicates that EAI may be used as an early predictor of grain yield.

The slope of the relationship between $S_{e}$ and the grain dry mass per ear shows large genotypic variability (Fig. 10), which can be explained by differences between varieties in ear length (Fig. S10a) but not in ear compactness (Fig. S10b). These results suggest that an allometric relationship exists between $S_{e}$ and grain dry mass per ear, which could be exploited to dissect the effect of environmental conditions in yield components through optical observations in the field.

## 5 Conclusion

In this study we present a novel methodology based on RGB images and LiDAR point clouds taken by the Phenomobile V2 ground robot to estimate $D_{e}$ and $S_{e}$ of field grown wheat crops at high throughput. The methodology was evaluated in three field trials where ten elite bread wheat varieties were grown under different environments (sites, years, irrigation, sowing density, and sowing dates). The estimation of $D_{e}$ from ear detection with the YOLOv5 CNN presented in this paper differs from methodologies of existing studies in two ways, the use of flashes during image acquisition to maximize the visibility of ears in vertical photographs, and the possibility to compute accurately the image footprint from a LiDAR mounted next to the camera. Thanks to these improvements, the relative error of $D_{e}$ estimations was reduced compared to previous studies.

To estimate $S_{e}$ a new method was developed, based on the inversion of the Beer-Lambert law, adapted to compute the extinction coefficient for ears and using as input the ear gap fraction estimated from RGB image segmentation. Our method provides satisfactory results, with relative errors against destructive measurements below 20% for awnless varieties. Interestingly, the results demonstrated that destructive measurements of $S_{e}$ for awned varieties using a planimeter are biased and that our automatic method provides more accurate estimations of $S_{e}$.

The methods developed to estimate $D_{e}$ and $S_{e}$ rely on α and AEA parameters, respectively. The value for these parameters derived in this study from field measurements should be valid for other phenotyping instruments acquiring canopy RGB images at nadir and at 45°. Further works should verify this point.

Phenomobile estimations of EAI and $S_{e}$ were strongly correlated with grain yield and grain dry mass per ear, respectively. This suggests that $S_{e}$ may constitute a proxy for important yield components. We analysed the variability of both traits due to G × E interactions and identified varieties with systematically larger ears and showed that $S_{e}$ is reduced in late winter sowings probably due to a shortening of the stem elongation phase. These differences in $S_{e}$ are, according to our analysis, due to changes in the width of the ears, and not their length. The same analysis on $D_{e}$ allowed us to quantify the influence of the initial sowing density, the sowing date, and the water stress. Future studies should further assess the potential use of $D_{e}$ and $S_{e}$ estimations from RGB and LiDAR data to monitor G × E interactions for yield components.

## Funding

This study was funded by the ANR (French National Research Agency) as part of the FFAST (Functioning from the Assimilation of Structural Traits) project (grant number ANR-21-CE45-0037) and the Programme d’Investissements d'avenir PHENOME (grant number ANR-11-INBS-0012).

## Author contribution

R.L.L. and P.M.: conceived the research and the trials. M.P.D.A.: developed the method, analysed the data and wrote the first draft of the paper. R.L.L. and S.J.: contributed to the development of the method. B.S. developed the ear segmentation algorithm from RGB images. A.G., B.B., R.C., R.L-R., R.M, M.R, G. T., V. M. and F.V: conducted the field trials. F.L., A.A. and P.B.: validated and curated field data. All reviewed and edited the manuscript.

The authors are grateful with Andreas Hund (ETH, Zurich) for his support in the statistical analysis of multi-environmental trials.

## Data availability

The algorithm developed to estimate the *EAI* and the average ear surface using binary images from ear segmentation are publicly available in the repository https://forge.inrae.fr/raul.lopez-lozano/wheat-ear-surface, jointly with an example dataset from the Mauguio 2023 trial (4 treatments, 1 replicate). The Phenomobile estimations of ear surface area and ear density used in the multi-environmental mixed model presented in Section 2.5 are included as supplemental material.

Any other additional data used in this study are available from the corresponding author upon reasonable request.

## Declaration of competing interest

The authors declare that they have no known competing financial interests or personal relationships that could have appeared to influence the work reported in this paper.
