# Supplementary material for: High-throughput phenotyping of wheat ear surface area and ear density in the field
Source: Plant Phenomics. 2026 Mar 9;8(2):100199. doi: 10.1016/j.plaphe.2026.100199 (PMC13316381; doi:10.1016/j.plaphe.2026.100199)
Supplement: Multimedia component 2 [file mmc2.docx]

# Supplementary Materials

High-throughput phenotyping of wheat ear surface area and ear density in the field

Marie-Pia D’Argaignon, Raul Lopez-Lozano, Sylvain. Jay, Auréilien Ausset, Bruno Berthon, Philippe Burger, Romain Chapuis, Benoît de Solan, Antonin Grau, Florian Larue, Romaine Le-Roy, Rémy Marandel, Vincent Mercier, Mathieu Roy, Gilles Tison, Frédéric Venault, and Pierre Martre

| **Table S1. Registration year, cold requirement, precocity at booting and heading, plant height, and awnedness of the 10 varieties used in this study. Values of cold requirement, precocity at booting and heading and plant height are expressed on a scale ranging from 1 to 9. Data were obtained from** [**http://www.fiches.arvalis-infos.fr.**](http://www.fiches.arvalis-infos.fr.) | | | | | | |
| --- | --- | --- | --- | --- | --- | --- |
| **Variety name** | **Registration year** | **Cold requirement^a^** | **Precocity at booting^b^** | **Precocity at heading^b^** | **Plant height^c^** | **Awnedness** |
| Renan | 1990 | 1 | 1 | 6 | 4 | Awned |
| Fructidor | 2014 | 2 | 3 | 6 | 3.5 | Awnless |
| Rubisco | 2012 | 3 | 3 | 6.5 | 3 | Awned |
| Nemo | 2015 | 3 | 3 | 6.5 | 3.5 | Awned |
| LG Absalon | 2016 | 3 | 3 | 6.5 | 3.5 | Awnless |
| Chevignon | 2017 | 3 | 2 | 6 | 4 | Awnless |
| Apache | 1998 | 4 | 3 | 7 | 3.5 | Awnless |
| RGT Sacramento | 2014 | 4 | 6 | 6.5 | 3.5 | Awned |
| Oregrain | 2012 | 5 | 4 | 7 | 3.5 | Awnless |
| Nogal | 2006 | 8 | 5 | 8 | 3.5 | Awned |
| ^a^ 1: very winter, 9: true spring.  ^b^ 1: very early, 9: very late.  ^C^ 1: very short, 9: very tall. | | | | | | |

| Table S2. Dates of the Phenomobile V2 RGB and LiDAR observations at anthesis (GS65), early dough (GS83), and maturity (89) in the three trials considered in this study for the estimation of ear density and ear surface area. | | | | |  |
| --- | --- | --- | --- | --- | --- |
| Trial | **Sowing treatments^a^** | **Phenomobile acquisition dates** | | | |
|  |  | ***G665*** | ***GS83*** | ***GS89*** | |
| DiaPhen 2022 | S1 | 2022-05-09 | 2022-06-01 | 2022-06-13 | |
|  | S2 | 2022-05-23 | 2022-06-17 | 2022-06-24 | |
| DiaPhen 2023 | S1 | 2023-05-03 | 2023-05-30 | 2023-06-12 | |
|  | S2 | 2023-05-18 | 2023-06-07 | 2023-06-16 | |
| AgroPhen 2023 | S1 | 2023-05-08 | 2023-06-05 | 2023-06-16 | |
|  | S2 | 2022-05-15 | 2023-06-05 | 2023-06-23 | |
| ^a^ S1, autumn sowing; S2, winter sowings. | | | | |  |

| Table S3. Correlation matrix of grain yield, thousand kernel weight (TKW), grain number per year, and ear density ($\boldsymbol{D}_{\boldsymbol{e}}\boldsymbol{)}$ per trial and across trials. Data are Pearson’s correlation coefficients. P-values are given in parenthesis. Statistically significant correlations (P < 0.05) are in bold font. | | | | | |
| --- | --- | --- | --- | --- | --- |
| AgroPhen 2023 | | | | | |
|  | **Grain yield** | **TKW** | **Grain number per ear** | | $\boldsymbol{D}_{\boldsymbol{e}}$ |
| Grain yield | 1 | -0.08(0.54) | **0.38** | | **0.54** |
| TKW |  | 1 | **-0.45** | | -0.13(0.32) |
| Grain number per ear |  |  | 1 | | **-0.41** |
| $\boldsymbol{D}_{\boldsymbol{e}}$ |  |  |  | | 1 |
| DiaPhen 2022 | | | | | |
|  | **Grain yield** | **TKW** | **Grain number per ear** | | $\boldsymbol{D}_{\boldsymbol{e}}$ |
| Grain yield | - | **0.85** | **0.46** | | **0.33** |
| TKW |  | - | 0.12(0.26) | | 0.1(0.34) |
| Grain number per ear |  |  | - | | -0.13(0.25) |
| $\boldsymbol{D}_{\boldsymbol{e}}$ |  |  |  | | - |
| DiaPhen 2023 | | | | | |
|  | **Grain yield** | **TKW** | **Grain number per ear** | | $\boldsymbol{D}_{\boldsymbol{e}}$ |
| Grain yield | - | **0.8** | **0.64** | | **0.74** |
| TKW |  | - | **0.29** | | **0.41** |
| Grain number per ear |  |  | - | | **0.24** |
| $\boldsymbol{D}_{\boldsymbol{e}}$ |  |  |  | | - |
| Across trials | | | | | |
|  | **Grain yield** | **TKW** | **Grain number per ear** | $\boldsymbol{D}_{\boldsymbol{e}}$ | |
| Grain yield | - | **0.71** | **0.51** | **0.44** | |
| TKW |  | - | 0.04(0.5) | **0.26** | |
| Grain number per ear |  |  | - | **-0.14** | |
| $\boldsymbol{D}_{\boldsymbol{e}}$ |  |  |  | - | |


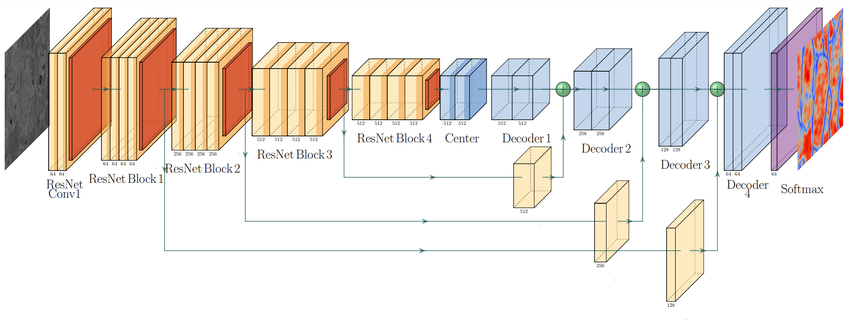


**Fig. S1.** Unet-ResNet18 architecture. From Fulton et al., (2024)


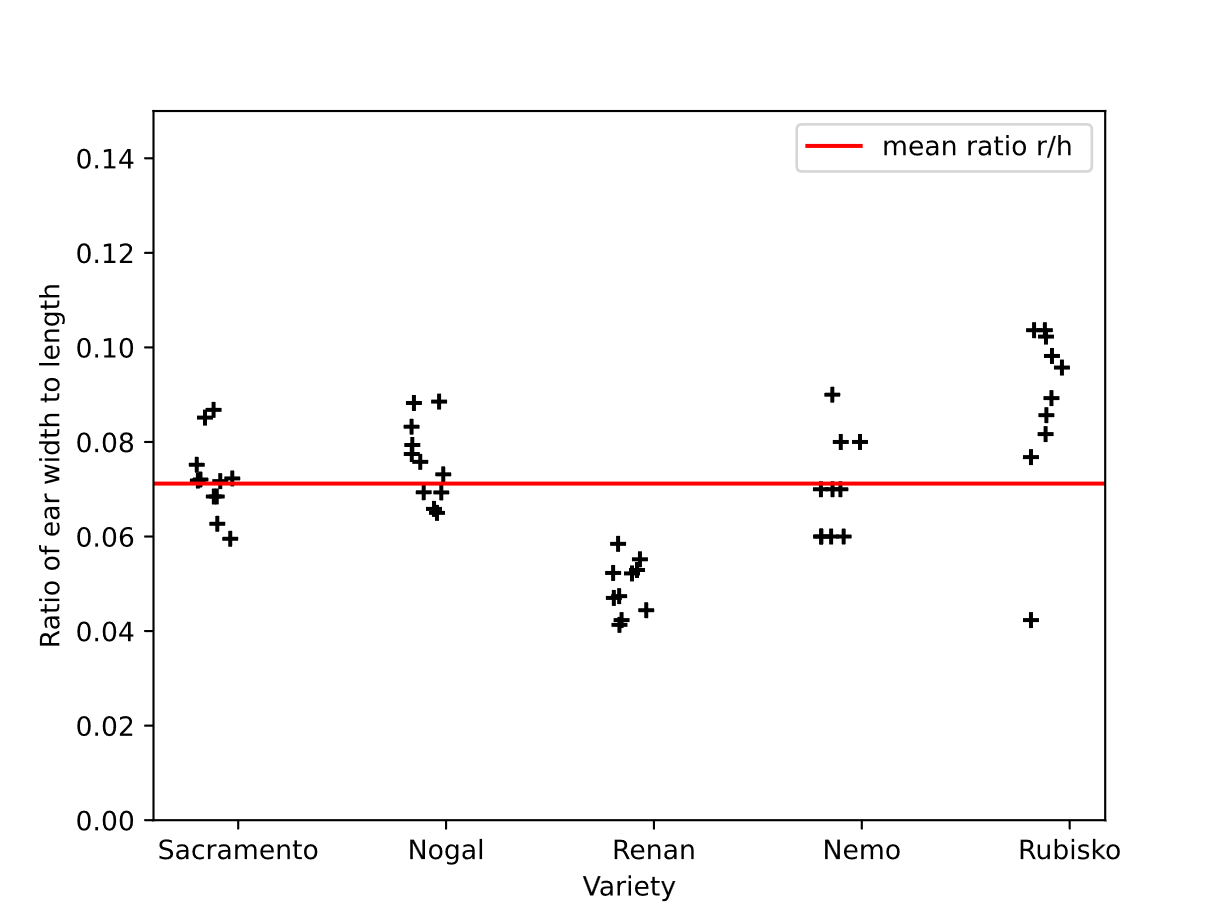


**Fig. S2.** Jitter plot of the ratio of ear width to length ($r/h$) five winter wheat awned varieties measured in the AgroPhen 2023 trial in the winter sowings treatment. Ear width and length were measured destructively using calliper on 10 ears per variety. Data for individual ears are shown.


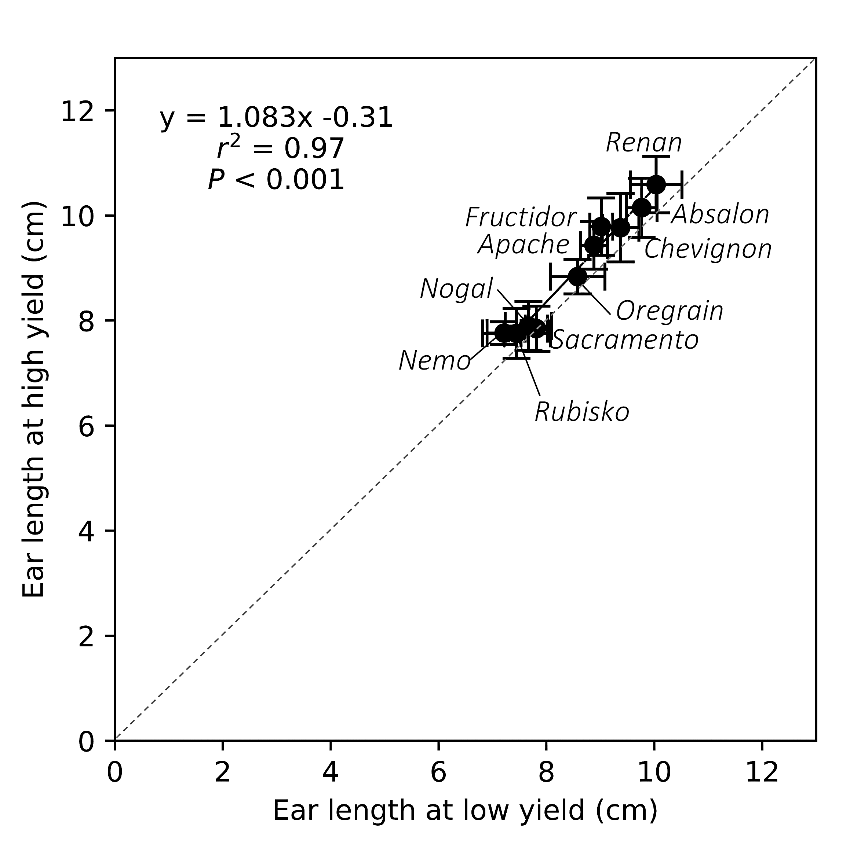


**Fig. S3.** Relationship between the ear length per variety measured at harvest in high yield and low yield environments. Ear length was measured manually on 20 ears per variety and environment. High yield environments: Agrophen 2023 (winter sowings, autumn sowings) and DiaPhen autumn sowings (2022 and 2023). Low yield environments: DiaPhen winter sowings (2022 and 2023). Error bars are the standard deviations across treatments. The dashed line is the 1:1 line.


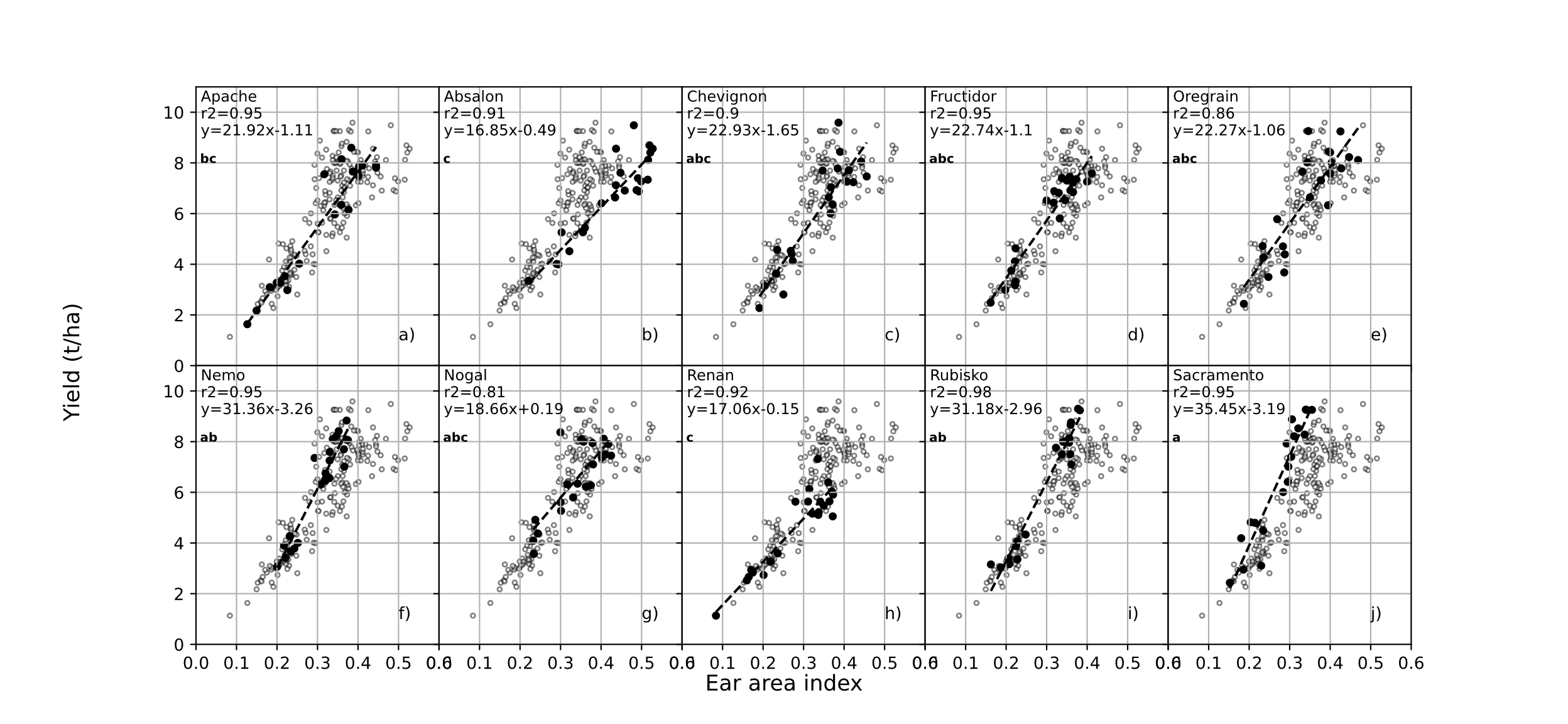


**Fig. S4.** Relationship between yield and estimated EAI from the Phenomobile V2 for the 10 variety used in this study (black circles). (a – e) Awnless varieties. (f – j) Awned varieties. Data are the values measured or estimated for single microplots in the three trials considered in this study (n = 213). In total, 27 microplots were affected by lodging and not taken into account. Lines are linear regression (all P < 0.01) fitted to each variety. The empty circles represent data for all 10 varieties shown to facilitate the visual comparison of the varieties. Letters in bold indicate statistically significant differences in the slope between varieties at 95% confidence interval.


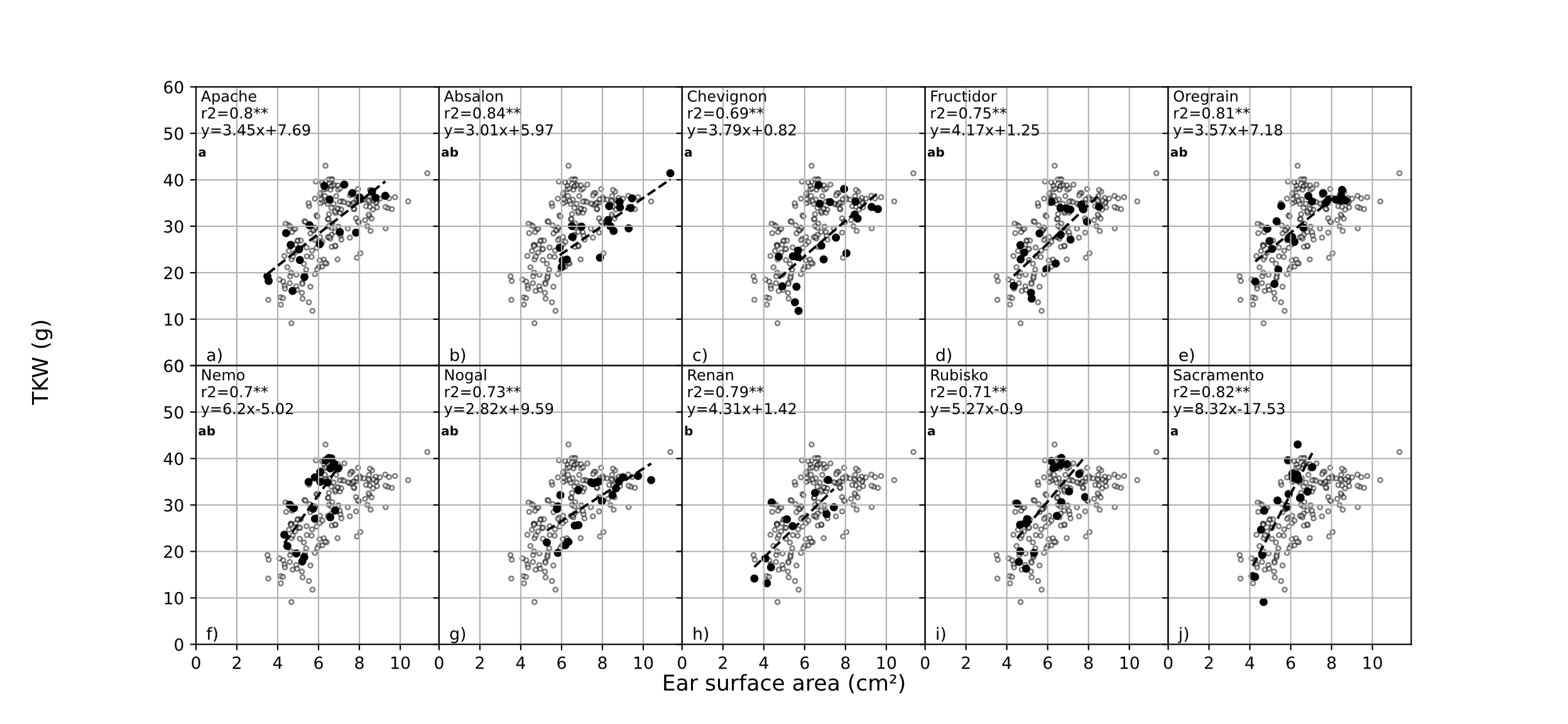


**Fig. S5.** Genotype-specific relationship between the estimated surface area from Phenomobile RGB images and the thousand kernel weight. On each graph, the values correspond to the measurements and estimation on individual microplots for the DiaPhen 2022, DiaPhen 2023 and AgroPhen 2023 trials. Lines are linear regression (all P < 0.01) fitted to each variety. The empty circles represent data for all 10 varieties shown to facilitate the visual comparison of the varieties. n = 213. In total, 27 microplots were affected by lodging and not included.


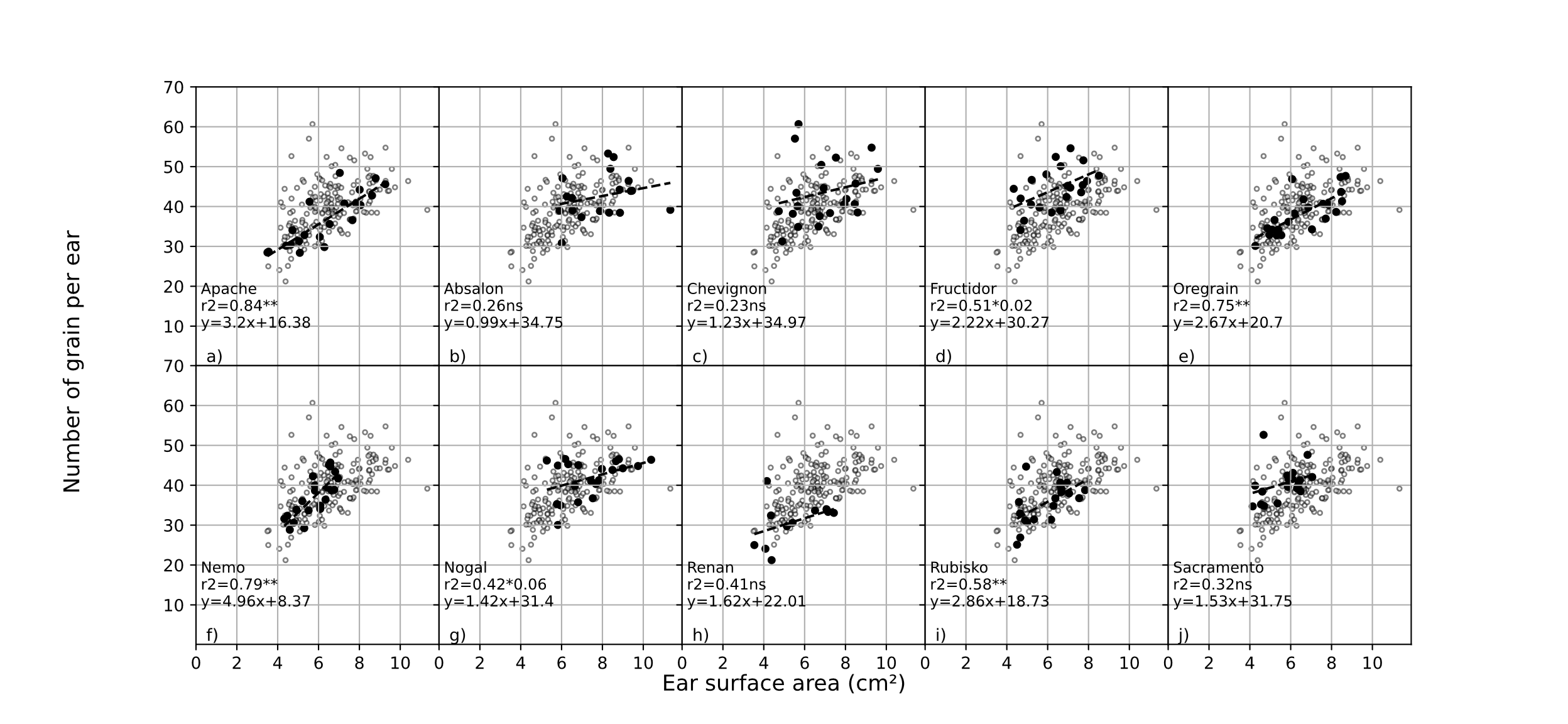


**Fig. S6.** Genotype-specific relationship between the estimated surface area from Phenomobile RGB images and and number of grains per ear. On each graph, the values correspond to the measurements and estimation on individual microplots for the DiaPhen 2022, DiaPhen 2023 and AgroPhen 2023 trials. The empty circles represent data for all 10 varieties shown to facilitate the visual comparison of the varieties. n = 213. In total, 27 microplots were affected by lodging and not included. * indicate P<0.05, ** indicate P < 0.01, ns indicate P > 0.05.


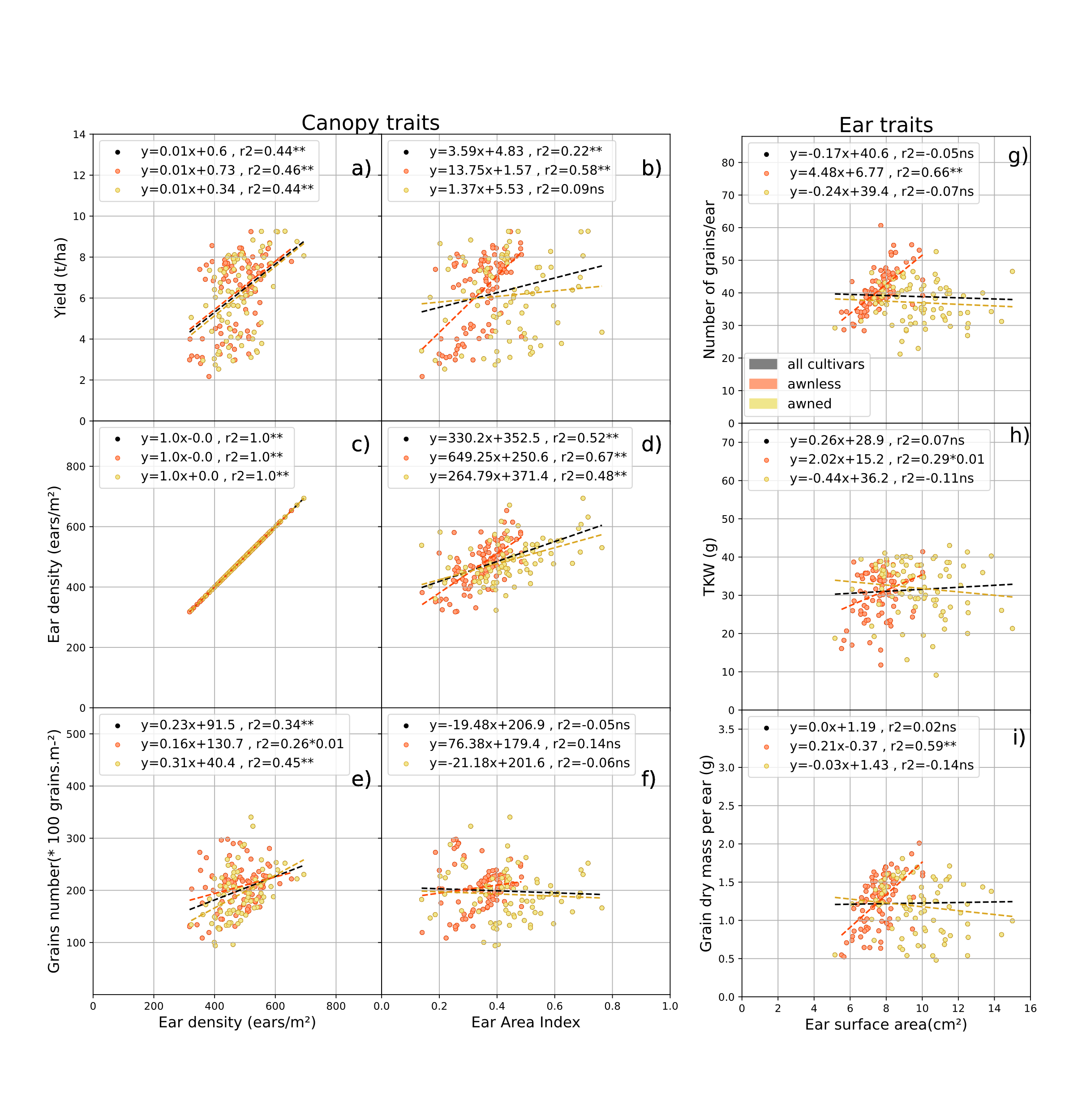


**Fig. S7.** Relationship between yield components and traits measured manually at harvest across sites and treatments. At the canopy scale the correlations between yield (a-b), ears density (c-d) and grains number per m2 (e-f) with ear density (a,c,d) and ear area index EAI (b,d,f) are shown. At the ear scale the correlation between the ear surface area with grain dry mass per ear (i), number of grains per ear (g) and thousand kernels weight (h) are presented. Each point represents the value measured or estimated for a single microplot. Lines are linear regression (all P < 0.01) fitted to the awnless (red lines), awned (yellow lines), and all varieties (black lines). On all graphs n = 169. **, P < 0.01; *, 0.01 < P < 0.05; n.s., P > 0.05.


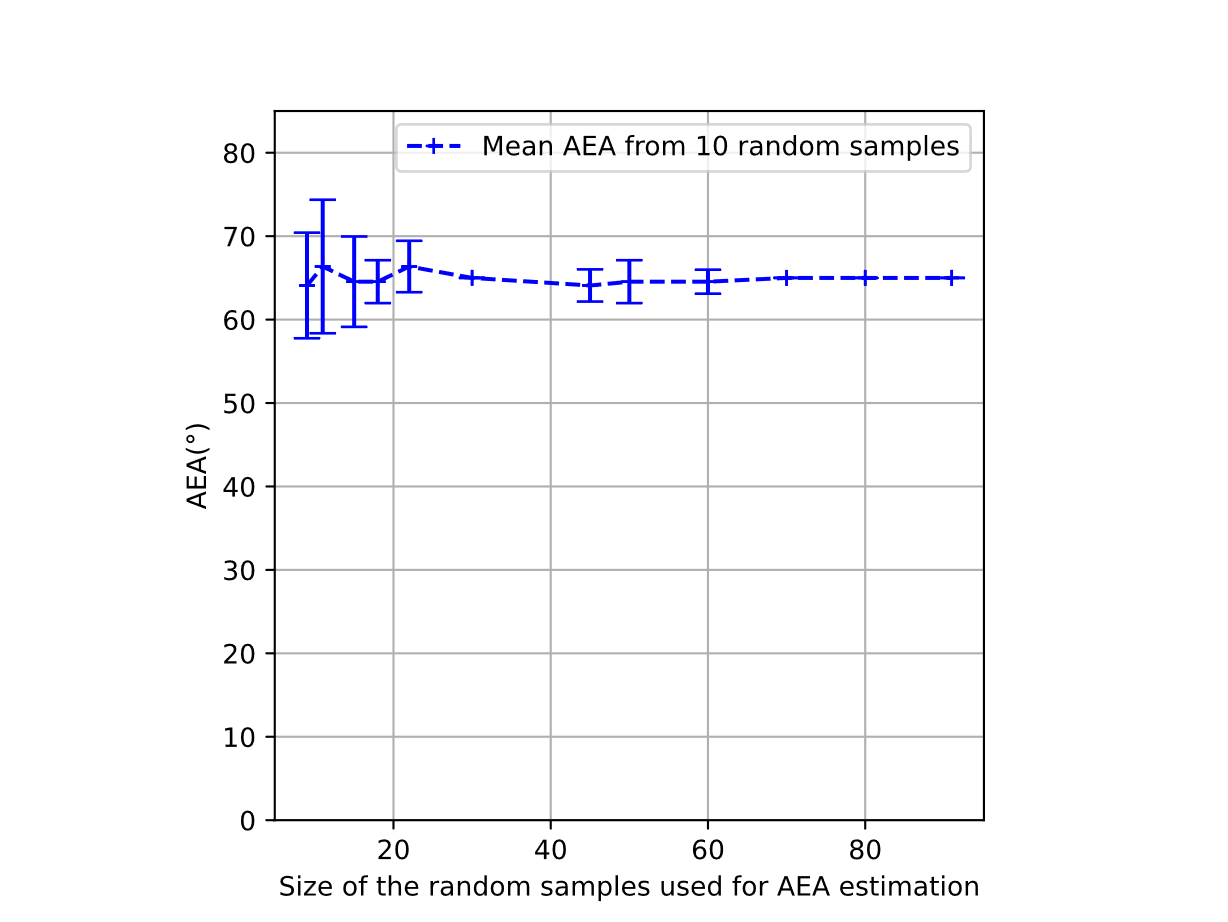


**Fig. S8.** Nominal AEA value at BBCH83, estimated using subsamples of different sizes drawn from all plots of the awnless varieties. For each subsample size, the optimal AEA was computed ten times, corresponding to ten independent random selections. The mean and standard deviation of these ten repetitions are shown using dotted lines and error bars, respectively.


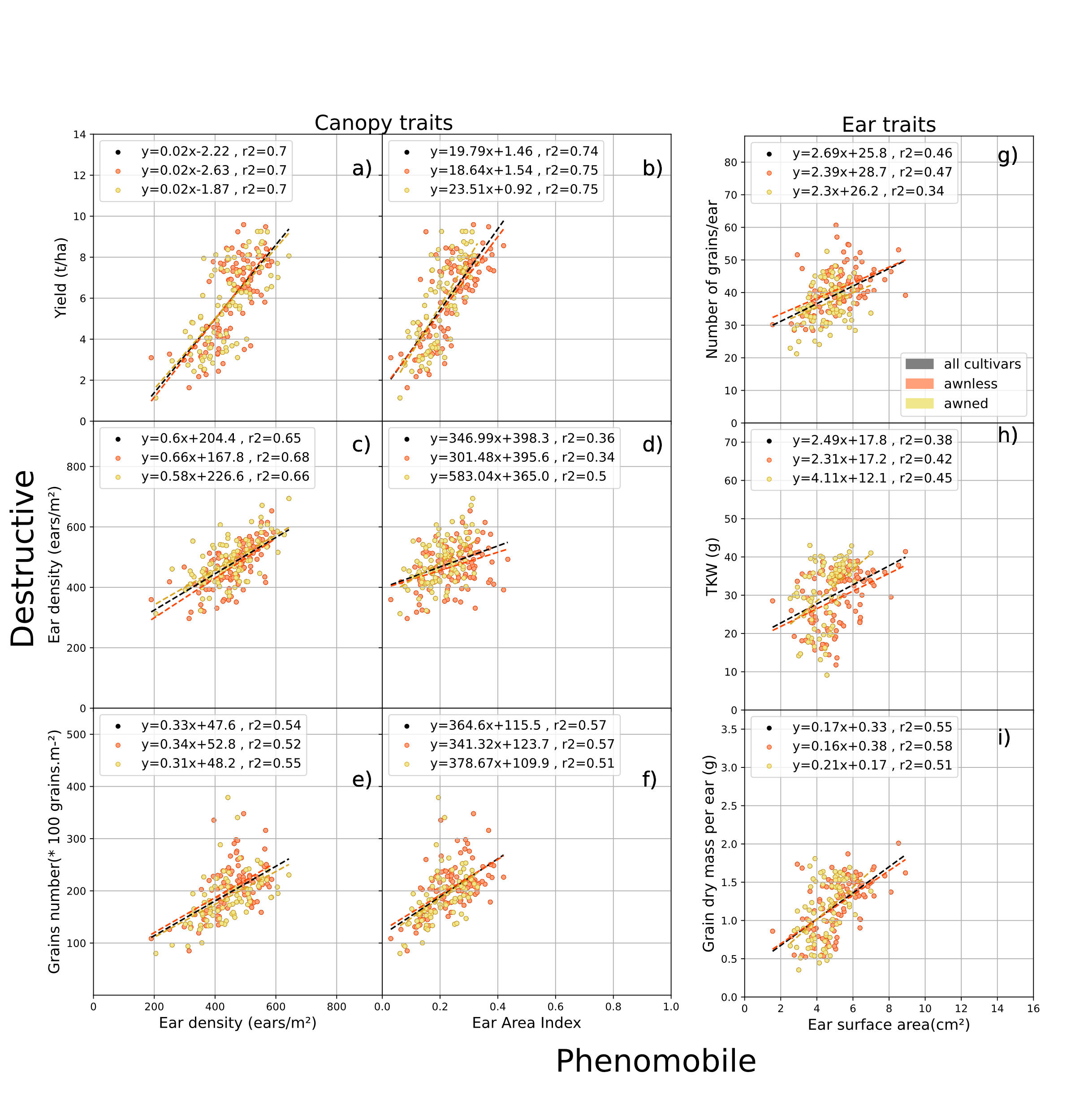


**Fig. S9.** Relationship between yield components and traits estimated from the Phenomobile V2 at growth stage GS65 (AEA = 85°). (a – f) Grain yield (a, b), ear density (c, d) or grains number per m2 (e, f) versus ear density (a, c, d) or ear area index (EAI; b, d, f). (g – i) Grain dry mass per ear (i), number of grains per ear (g) and thousand kernels weight (h) versus ear surface area. Data are the values measured or estimated for single microplots (n = 213). In total, 27 microplots were affected by lodging and not included. Lines are linear regression (all P < 0.01) fitted to the awnless (red lines), awned (yellow lines), and all varieties (black lines).


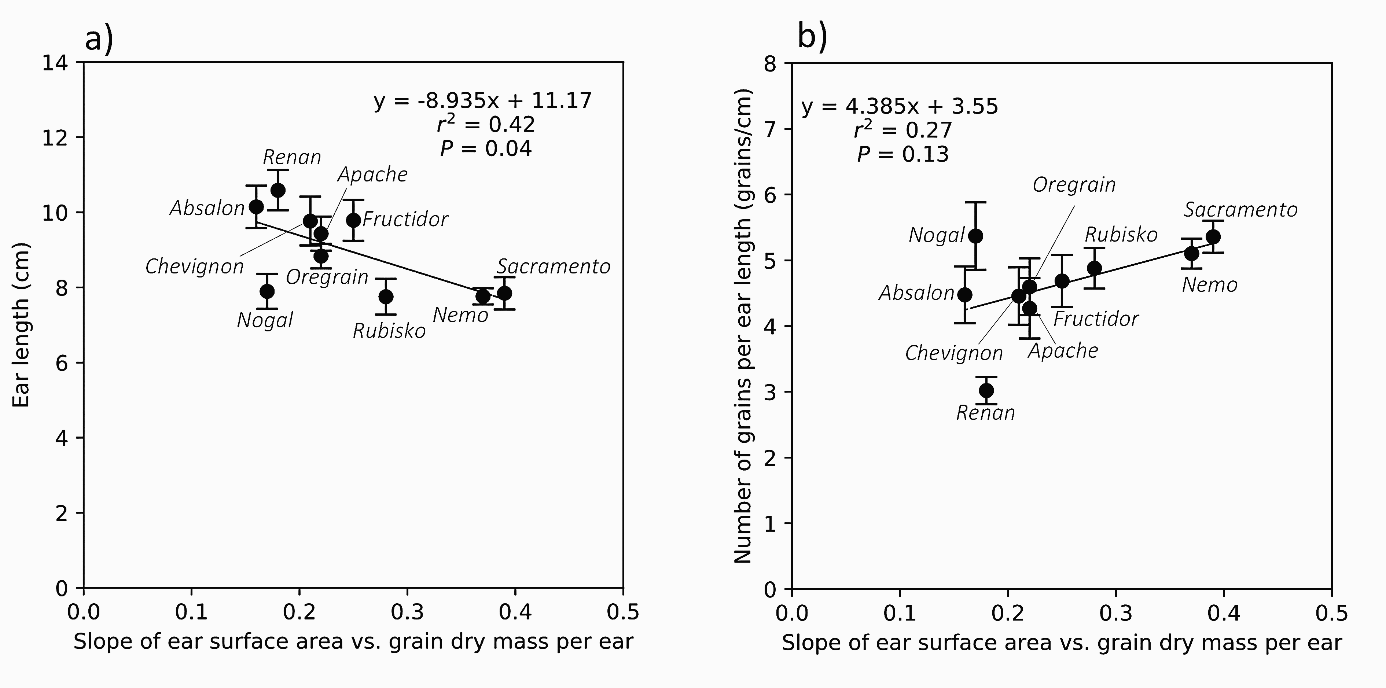


**Fig. S10.** Relationship between the average ear length (a) and the number of grains per ear length (b) and the slope of the linear model between the average ear surface area and grain dry mass per ear (see Fig. 10 in the main text). Data are means ± 1 s.d. across plots in high yield environments: Agrophen 2023 (winter sowings, autumn sowings) and DiaPhen autumn sowings (2022 and 2023).
